# Global copy number analyses by next generation sequencing provide insight into pig genome variation

**DOI:** 10.1186/1471-2164-15-593

**Published:** 2014-07-14

**Authors:** Jicai Jiang, Jiying Wang, Haifei Wang, Yan Zhang, Huimin Kang, Xiaotian Feng, Jiafu Wang, Zongjun Yin, Wenbin Bao, Qin Zhang, Jian-Feng Liu

**Affiliations:** National Engineering Laboratory for Animal Breeding, Key Laboratory of Animal Genetics, Breeding and Reproduction, Ministry of Agriculture, College of Animal Science and Technology, China Agricultural University, Beijing, 100193 China; Shandong Provincial Key Laboratory of Animal Disease Control and Breeding, Institute of Animal Science and Veterinary Medicine, Shandong Academy of Agricultural Sciences, Jinan, 250100 China; Virginia Bioinformatics Institute, Virginia Tech, Washington Street, Blacksburg, Virginia MC0477, 24061 USA; School of Animal Science, Guizhou University, Guiyang, 550025 China; College of Animal Science and Technology, Anhui Agricultural University, Hefei, 230036 China; College of Animal Science and Technology, Yangzhou University, Yangzhou, 225009 China

**Keywords:** Copy number variations (CNVs), Segmental duplications (SDs), Next generation sequencing (NGS), Pigs

## Abstract

**Background:**

Copy number variations (CNVs) confer significant effects on genetic innovation and phenotypic variation. Previous CNV studies in swine seldom focused on in-depth characterization of global CNVs.

**Results:**

Using whole-genome assembly comparison (WGAC) and whole-genome shotgun sequence detection (WSSD) approaches by next generation sequencing (NGS), we probed formation signatures of both segmental duplications (SDs) and individualized CNVs in an integrated fashion, building the finest resolution CNV and SD maps of pigs so far. We obtained copy number estimates of all protein-coding genes with copy number variation carried by individuals, and further confirmed two genes with high copy numbers in Meishan pigs through an enlarged population. We determined genome-wide CNV hotspots, which were significantly enriched in SD regions, suggesting evolution of CNV hotspots may be affected by ancestral SDs. Through systematically enrichment analyses based on simulations and bioinformatics analyses, we revealed CNV-related genes undergo a different selective constraint from those CNV-unrelated regions, and CNVs may be associated with or affect pig health and production performance under recent selection.

**Conclusions:**

Our studies lay out one way for characterization of CNVs in the pig genome, provide insight into the pig genome variation and prompt CNV mechanisms studies when using pigs as biomedical models for human diseases.

**Electronic supplementary material:**

The online version of this article (doi:10.1186/1471-2164-15-593) contains supplementary material, which is available to authorized users.

## Background

Copy number variations (CNVs) distribute ubiquitously in the human genome [1, 2] and belong to the spectrum of genetic variation ranging from 50 base pairs to larger structural events [3]. As an important form of genetic variation complementary to single-nucleotide polymorphisms (SNPs), CNVs have attracted extensive attentions and unprecedented successes have been achieved in detection of CNVs as well as segmental duplications (SDs) in the human genome [4–7]. Multiple studies indicated that CNVs have been associated with a variety of human diseases [8–12]. Together with SNPs, CNVs are becoming recognized as an important source of genetic variance [13] and may account for some of the missing heritability for complex traits [14].

Benefitting from the achievements of pioneering CNV studies in humans, substantial progress has been made in the discovery and characterization of CNVs in livestock genomes. In the past few years, a significant amount of research on genome-wide CNV identification was conducted in various domestic animal species, including cattle [15, 16], dog [17–19], sheep [20], goat [21], chicken [22], turkey [23] and pig [24, 25]. A suite of genes with copy number alteration were exploited contributing to variation of either Mendelian phenotypes [26–28] or complex production traits [29]. Based on these findings, it was expected that CNV studies could advance the studies of genetic diversity, evolution, functional genomics as well as genome assisted prediction.

However, a potential issue with majority of previous CNV studies in livestock species displayed as a lack of power and accuracy for CNV identification due to the technical limitations of two most frequently used detection platforms, i.e., SNP chips and array comparative genome hybridization (aCGH) [3, 6, 15, 30]. This obviously highlights the need to pursue more powerful and sensitive tools for construction of high resolution CNV map. To achieve this goal, Bickhart *et al.* [15] performed CNV detections in individual cattle genomes using the next-generation sequencing (NGS) technique combined with mrFAST/mrsFAST and whole-genome shotgun sequence detection (WSSD) analytical methods [5, 6, 31] based on the findings of SD detection [32]. Their work demonstrated that the NGS has superiority over SNP chip and aCGH in CNV deteciton in livestock genomes. Besides the platforms employed in CNV detection, the other crucial factor determining the abundance of detected CNV is the experimental population investigated. Findings from several studies [17, 24, 33] indicated that a considerable proportion of CNVs likely segregate among distinct breeds, such that a sufficiently high-resolution CNV map would require the survey of multiple breeds/populations [34].

In the past few years, much effort has been taken to detect CNVs in pig genome using three main genome-wide CNV identification technologies, i.e., aCGH [35–37], SNP genotyping array [24, 25, 38–40] and genome re-sequencing based on the next generation sequencing [41–43]. However, compared to humans and other model organisms, relatively few studies have investigated CNVs in pigs and little is known about how CNVs contribute to normal phenotypic variation and to disease susceptibility in this species. Since CNVs play a vital role in genomic studies, and pigs act as one of the most economically important livestock worldwide as well as popular model for various human diseases [44], it is an imperative need to develop a comprehensive, more accurate and higher resolution porcine CNV map and in-depth characterize CNVs across pig genomes for follow-up CNV functional investigation. To achieve the aforementioned goal, we performed the current study to systematically exploit features of SDs and CNVs present in the pig genome using high throughput NGS data of diverse pig breeds in the framework of the pig draft genome sequence (Sscrofa10.2) [45]. We designed the studies considering the following two aspects: (1) CNVs mostly occurred with different probabilities among different populations; and (2) A number of Chinese local breeds conferred much larger variability and higher average heterozygosity than European breeds [46].

Beyond the definition of CNVs, some CNVs may be fixed in the population and (if they are in state of gain) can also be detected across the genome as SDs [47] which are generally defined as >1 kb stretches of duplicated DNA with 90% or higher sequence identity [48]. It was also believed that an SD-rich region would generate more CNVs than other regions [48], showing a close association with CNVs near or around it. Considering the potential link between SDs and CNVs across the genome, we employed the NGS data of genomes of experimental individuals as well as the reference genome of Duroc 2–14 to construct individualized SD and CNV maps and in-depth characterize global CNVs via the commonly used analytical approaches, i.e., whole-genome assembly comparison (WGAC) and whole-genome shotgun sequence detection (WSSD) [6, 7, 49].

To pursue a reliable CNV map, in the present study, we employed individual genomes from multiple populations, including all six types of Chinese indigenous breeds, one Asian wild sow, as well as three commercial breeds. Additionally, we have improved the original read depth (RD) method in WSSD analyses through adjusting the bias in CNV calling due to fragmented sequences in the process of hard masking of reference genome. This enhanced the detection power, lowered the false positive findings and increased copy number estimation accuracy, especially for NGS data with long sequencing reads. Our work is of importance to researchers working with swine genomics and would lay a solid foundation for future CNV functional researches in the pig genome.

## Results

### Sequencing data set statistics

Based on Illumina HiSeq 2000, we obtained NGS data of 13 pig individuals, which were selected to cover a broad representation of pig diversity of both modern commercial pigs and Chinese domestic and wild pigs. The sequencing data set statistics have also been summarized in Table 1. The depth of coverage for each animal varied from 10.4× to 17.4×, which is sufficient for genome-wide CNV detection using RD method according to the previous studies [5, 6, 15].

**Table 1:** **The sequencing dataset statistics of the 13 analyzed pigs**

Sample name	Breed	Sex	# of raw reads	Raw depth of coverage	Breadth of coverage (%) ^a^	# of mapped reads	# of total mapping	Portion of mapped reads (%)	Average mapping count per read
A1	Asian wild population	Female	425059598	15.14	98.34	77911417	89797365	18.3	1.15
C3	Landrace	Female	299035346	10.65	98.67	54229309	62204847	18.1	1.15
D4	Duroc	Female	292290044	10.41	98.36	55895203	63458148	19.1	1.14
DN1	Diannan small-ear pig	Male	314388424	11.19	98.37	54588436	62544697	17.4	1.15
DN5	Diannan small-ear pig	Female	326384034	11.62	98.30	54440210	61948105	16.7	1.14
M2	Min pig	Female	335827092	11.96	98.29	61906969	71840511	18.4	1.16
MS7	Meishan pig	Female	311280060	11.08	98.38	52927456	60310779	17.0	1.14
MS8	Meishan pig	Female	327056954	11.65	98.37	57503480	65568705	17.6	1.14
R2	Rongchang pig	Male	489283828	17.42	98.37	84867123	96794730	17.3	1.14
W1	Daweizi pig	Female	319026072	11.36	98.31	55717064	63780180	17.5	1.14
Y2	Yorkshire	Female	310756334	11.06	98.52	57747078	66761766	18.6	1.16
Z2	Tibetan pig	Female	306511910	10.91	98.41	51705709	59208309	16.9	1.15
Z5	Tibetan pig	Female	306714914	10.92	98.68	55755070	64929977	18.2	1.16

^a^Calculation of covered percentage of genome is based on ungapped length of whole genome.

### SD map construction for the reference genome

Using WGAC, we initially detected a total of 902,068 pairwise alignments with an aligned length of >1 kb and identity of >90%, which showed an excess of SD contents compared to previous results in other species [32, 49, 50]. After removal of high-copy repeats, the filtered detections consisted of 28,509 pairwise alignments, of which 10,128 (35.5%) involved unplaced scaffolds (presented in Additional file 1: Table S1). Furthermore, 77.9% (22,214 of 28,509) of these alignments had an identity of >99% that may contain numerous artificial duplications due to local assembly errors [49]. The remaining alignments (6,295 of 28,509) had identities varying from 90% to 99%. The distribution profile of the identities for these 6,295 alignments was presented in Additional file 2: Figure S1, which showed an approximately uniform distribution within the interval of 0.90-0.98 while exhibiting a sharp increase in alignment frequency within the interval of 0.98-0.99. We further merged all of 28,509 alignments into 43,071 non-overlapping sequence intervals. The total length of these intervals reached 542.6 Mb, amounting to 19.3% of the reference genome, which indicated an excessive content of duplicated bases. Specially, 8,620 of 43,071 intervals were mapped to unplaced scaffolds, accounting for 121.0 Mb (57.1% of all the unplaced scaffolds). Among the 3,882 unplaced scaffolds >1 kb in size, 2,396 (61.7%) contained SD and 1,478 (38.1%) had >70% of duplicated bases (Additional file 2: Figure S2). The high content of SD in unplaced scaffolds was considered to be related to the difficulty in placing the scaffolds into the assembly [49].

**Figure 1:**
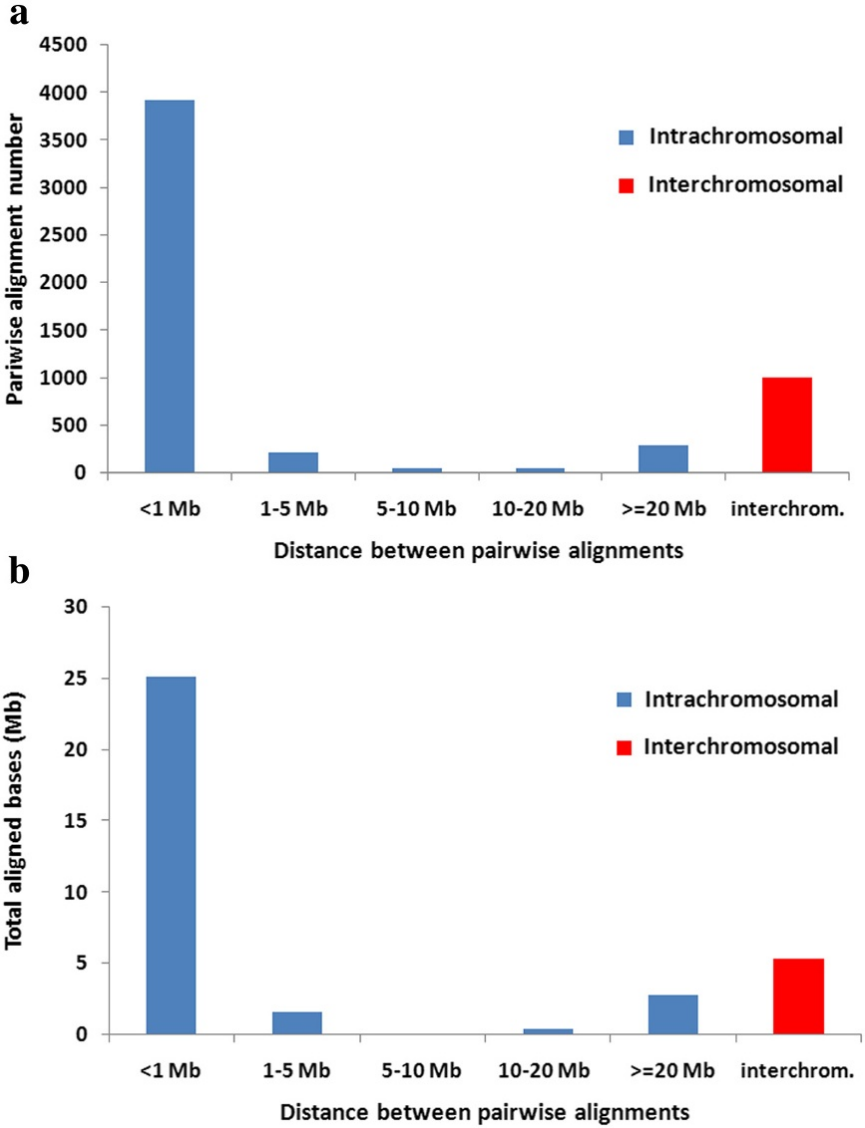
**Distribution of pairwise alignments within different distance ranges for SDs of the pig reference genome.** **(a)** The number of pairwise alignments of SDs varies from >3,500 to <100 against different distance ranges. **(b)** The total aligned bases of pairwise alignments against different distances varies from >25 Mb to <1 Mb. The total aligned bases is simply the sum of aligned bases of all pairwise alignments within different distances, probably counting multiple times for some regions covered by different pairwise alignments.

**Figure 2:**
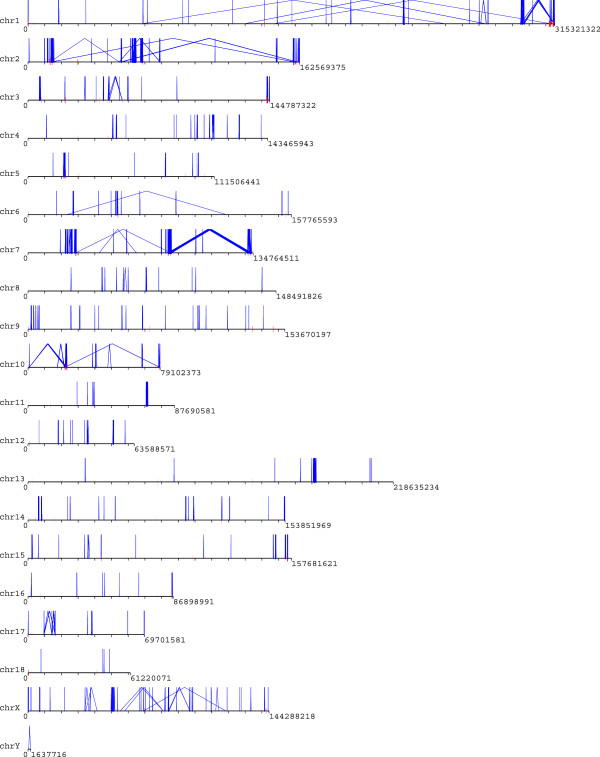
**Map of SDs (>5 kb) detected by WGAC method and filtered by WSSD results.** Intrachromosomal pairwise alignments are collected by blue line, and the interchromosomals are marked with short red lines. The map was drawn using the program parasight v7.6 (http://eichlerlab.gs.washington.edu/jeff/parasight/index.html).

In WSSD analyses, a total of 1,714 unique intervals (67.3 Mb) were predicted as listed in Additional file 1: Table S2. Similar to the strategy of Bailey *et al.* [7], we further filtered the WGAC alignments of ≥94% identity with SD calls by WSSD to remove artifactual duplications. After filtering, the final WGAC dataset consisted of 5,534 pairwise alignments (Additional file 1: Table S3), out of which 131 were mapped to unplaced scaffolds, and five were mapped to pig mitochondrion. Of the 20 chromosomes (1–18, X and Y), 4,529 of 5,398 (83.9%) pairwise alignments were intrachromosomal and most pairwise alignments were within the distance of 1 Mb between each other (Figure 1). The profile of the SD map with WGAC is presented in Figure 2 and the features of SDs across different chromosomes are also detailed in Table 2, which is similar to the duplication pattern of mouse [51], dog (22) and cattle [7, 18, 32, 51] while quite different from the interspersed segmental duplication pattern that predominates in human [7, 18, 32, 51]. Previous studies (8,47) suggested that abundant interspersed segmental duplications may be specific for human and great apes genomes and play a vital role during the evolution of their gene families.

**Table 2:** **SD distribution on different pig chromosomes**

Chr. name	Chr. length	Pairwise alignments		# of pairwise alignments within different distances						Length of pairwise alignments within different distances (bp) ^a^					
		#	Total length (bp)	<1 Mb	1-5 Mb	5-10 Mb	10-20 Mb	≥20 Mb	Interchrom.	<1 Mb	1-5 Mb	5-10 Mb	10-20 Mb	≥20 Mb	Interchrom.
1	315321322	591	3401742	222	32	2	39	26	270	2276538	246929	13940	295496	184744	1228308
2	162569375	704	5101992	507	2		1	32	162	3902938	20557		2826	689210	931033
3	144787322	295	2014549	136	1	9	2		147	1564008	4376	152465	11584		911669
4	143465943	287	1294886	235	6			1	45	1186413	49251			2684	111127
5	111506441	151	1053226	101	3				47	806564	148002				215744
6	157765593	386	1395733	215	3	1		21	146	1126648	14458	8377		97078	220477
7	134764511	796	5214940	457	63	3	1	166	106	3585639	1101644	22484	3239	2E + 06	847577
8	148491826	119	995588	66	6	2		1	44	853267	35624	8115		4117	98206
9	153670197	374	1888550	280				2	92	1595039				8290	311899
10	79102373	282	1981250	126	20	4	2	26	104	1262050	286602	18420	10717	395811	548851
11	87690581	135	999435	87	13				35	918600	151163				55653
12	63588571	165	803476	134	3	4			24	737632	14381	10696			49824
13	218635234	231	1238900	161			2	3	65	1080461			6220	11273	142648
14	153851969	351	1595288	289	1			1	60	1425665	2202			4606	168661
15	157681621	401	1640639	286	4			1	110	1458101	23471			9431	340589
16	86898991	332	620789	205					127	546490					163337
17	69701581	229	1089155	144	43	19		4	19	660648	434075	166802		19169	78385
18	61220071	65	275384	21					44	161453					130231
X	144288218	368	2254851	254	16	2	4	4	88	1847734	93124	22251	70387	66276	247951
Y	1637716	5	34635		2				3		28541				6094

^a^Pairwise alignments were merged into non-overlapping intervals which are used for measurements of the length.

The final pig SD database was constructed through integrating low-identity WGAC (<94%), filtered high-identity WGAC (≥94%) and the WSSD estimates. Overlapping segments by either WGAC or WSSD were simply merged into one single SD, the endpoints of which are outermost bases of the overlapping segments. Excluding unplaced scaffolds and mitochondrion, the pig SD database contained 2,860 intervals which totaled 73.5 Mb in size and 2.8% of all the chromosomes (1–18, X, Y) (Additional file 1: Table S4). The proportion of duplicated bases varied from 1.2% to 6.9% across different chromosomes as showed in Additional file 2: Figure S3. Compared to previous studies on other species [7, 18, 32, 51], the estimates of pig SD are relatively conservative. One possible reason may be due to exclusion of the unplaced scaffolds in our WSSD analysis.

### Individualized CNV discovery

Using our improved strategy, a total number of 13,517 segmental duplication/deletion calls were predicted from all the 13 individuals after artifact removal. The number of CNV events varied across different pig individuals, ranging from 870 (Yorkshire) to 1,311 (Duroc) with an average of 1,040 per individual (see Table 3). The overall profile of these identified segmental duplications/deletions across the genome for each individual is illustrated in Additional file 2: Figure S4, as well as detailed in Additional file 3: Table S5.

**Table 3:** **Summaries of SD/deletion calls of the 13 analyzed pigs on the number, total length and average length**

Sample	Number			Total length (Mb)			Mean size (kb)		
	# of total calls	# of duplications	# of deletions	All calls	Duplications	Deletions	All calls	Duplications	Deletions
A1	1064	763	301	48.5	40.9	7.6	45.6	53.7	25.2
C3	930	744	186	44.6	40.1	4.5	47.9	53.9	24.0
D4	1311	1130	181	48.8	44.8	3.9	37.2	39.7	21.8
DN1	951	684	267	45.3	38.9	6.4	47.6	56.9	23.8
DN5	1060	765	295	48.2	40.7	7.6	45.5	53.1	25.6
M2	1135	816	319	47.2	40.0	7.2	41.6	49.0	22.6
MS7	1052	795	257	49.6	43.6	6.0	47.1	54.8	23.4
MS8	958	711	247	44.9	38.5	6.4	46.9	54.2	25.9
R2	1099	766	333	44.7	37.2	7.5	40.7	48.5	22.6
W1	993	756	237	46.7	40.7	6.0	47.0	53.8	25.4
Y2	870	728	142	42.4	39.1	3.3	48.8	53.7	23.3
Z2	1025	753	272	46.2	39.7	6.5	45.0	52.7	23.9
Z5	1069	798	271	47.1	40.9	6.2	44.1	51.3	22.9
On average	1040	785	254	46.5	40.4	6.1	45.0	51.9	23.9

Accordingly, all detected CNV segments were further merged into 3,131 unique CNVRs across all experimental animal genomes following the criteria that the union of overlapping CNVs across individuals are considered as a CNVR [4]. Concerning copy number status, the numbers of gain, loss and both events (loss and gain within the same region) were 1702 (54.36%), 1366 (43.63%) and 63 (2.01%), respectively. Gain events were more common than loss events in CNVRs, and had slightly larger sizes than losses on average (36.15 kb vs. 23.99 kb). The CNVRs totaled 102.8 Mb in length with an average of 32.8 kb, amounting to 4.0% of the 20 chromosomes based on the porcine genome (*Sscrofa* 10.2). The distribution and the status of these identified CNVRs are plotted in Additional file 2: Figure S4, and a full list of CNVRs and corresponding features are provided in Additional file 3: Table S6. We further summarized the numbers and the lengths of CNVRs on different chromosomes in Additional file 3: Table S7, which illustrated non-uniform patterns across the genome. This is consistent with previous reports on heterogeneous distributions of CNVs in human and other species [4, 15].

**Figure 3:**
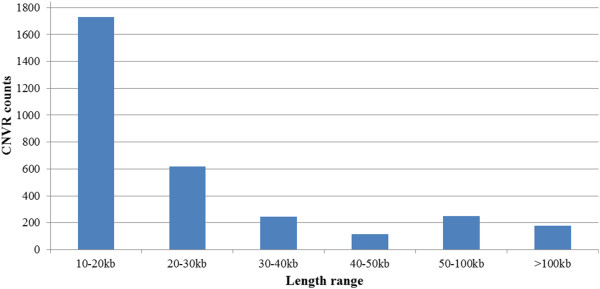
**The spectrum of the sizes of all detected CNVRs.**

Figure 3 demonstrates the spectrum of sizes of all detected CNVRs across the genome. It shows that most CNVRs fell into the interval between 10 kb and 20 kb, and the frequency of CNVRs tends to decrease with the increase of the length. It is notable that in our RD analyses, CNVs were called using the criterion that at least 6 of 7 sequential long sliding windows showed RD values significantly deviating from the RD average; thus, CNVs >10 kb in length were kept in the final dataset. This indicates that our RD analyses are prone to detection of large structural variation events, and a significant amount of variation in length <10 kb would be precluded from the final findings. This filtering process is a routine strategy in recent similar studies [5, 6, 15] to assure high confident positive findings in RD detection.

We investigated further to see if potential population/breed specific CNVs exist. Specifically, of the 3,131 total CNVRs, 1,679 (53.6%) were merely identified in a single breed/population, confirming that segregating CNVs exist across various breeds. Additionally, out of the 3,131 CNVRs, 612 (19.5%) were called merely in the three modern commercial breeds, while 1,513 CNVRs (48.3%) were detected specific in the nine Chinese indigenous pigs as well as the wild sow. These potential population/breed specific CNVRs can be considered as good candidates for determining breed-specific characteristics, although it is necessary to confirm phenotypic effects of these CNVs using more experimental samples. On the other hand, we scanned all CNVRs and merely found nine of them (4 duplications, 5 deletions) ubiquitously existing in the same state among the 13 animals. Except these nine potentially fixed SDs/deletions, the states of other SDs/deletions are variable across all 13 individuals. This clearly demonstrates CNVs are widely present in genomes across different population/breeds. We compared the length as well as the numbers of SDs/deletions identified between each pair of individuals. As given in Additional file 3: Table S8 and S9, the number of common intervals shared by the pairwise individuals ranges from 625 to 851, with the total overlapping length from 32.8 Mb to 40.1 Mb. This suggests that most CNVs occurred widely across the genomes of individuals.

### Quality assessment of CNVs by using aCGH data and qPCR

Using two complementary methods, aCGH and qPCR, we performed experimental validation to confirm individual copy number variants.

**Figure 4:**
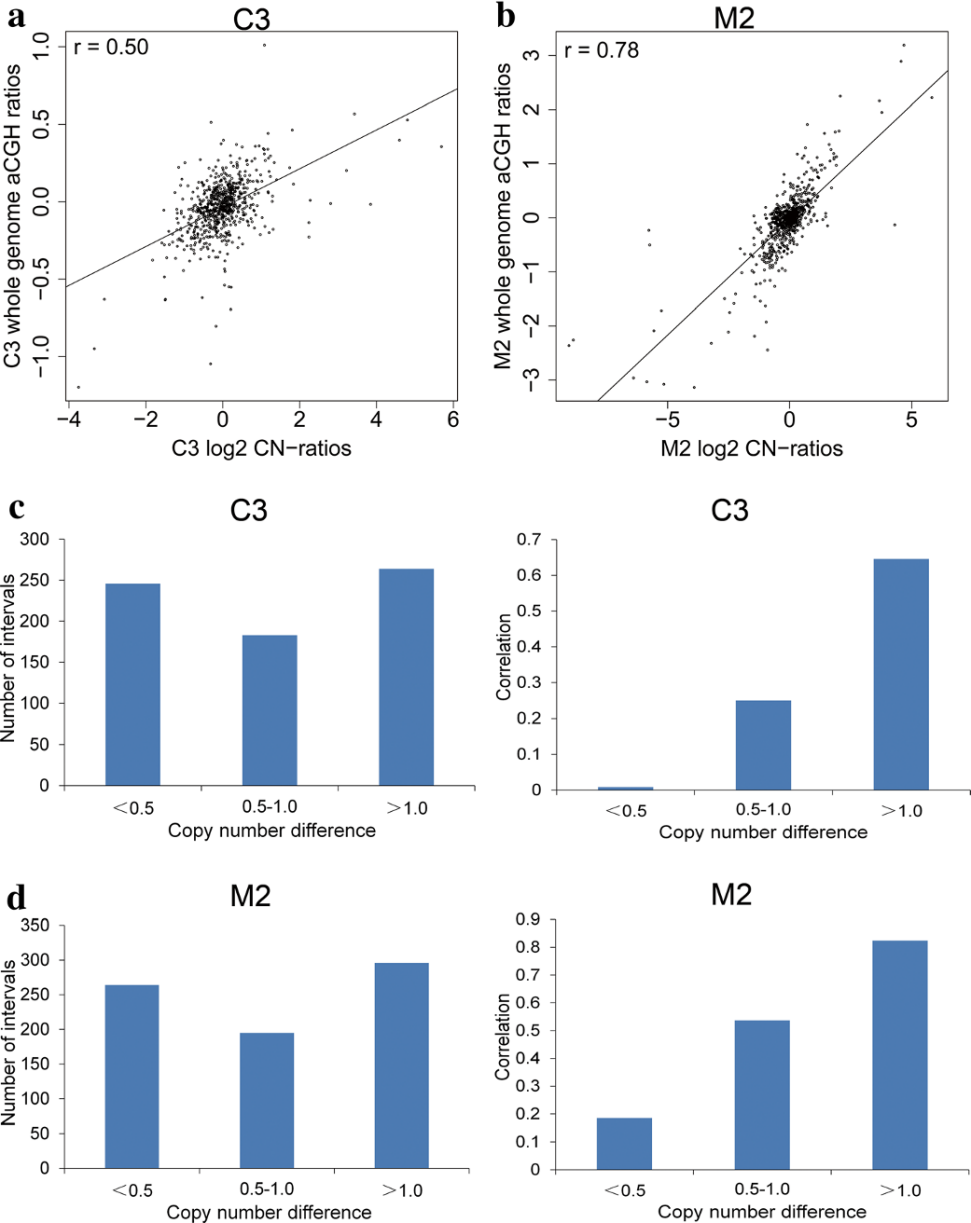
**The correlation between RD estimates and experimental aCGH results.** **(a)** For individual C3, the log_2_ values of ratios of RD predicted copy numbers (horizontal axis) were compared with probe log_2_ ratios from whole-genome aCGH (vertical axis), showing a correlation of 0.50.. **(b)** Another sample, M2, shows a correlation of 0.78. **(c)** For individual C3 (Landrace), CNV intervals were divided into three groups according to different level of copy number difference between C3 and the reference sample (D4, Duroc). Every group of CNV intervals was used to calculate the correlation between RD estimates and experimental aCGH results, respectively. It is clear that the higher the copy number difference of each interval, the larger the correlation between RD estimates and experimental aCGH results. **(d)** For another individual (M2, Min pig), impact of copy number difference on the correlation shows similar trend with that in Figure 3c.

One custom-designed 2.1 M aCGH (Roche-NimbleGen) based on the Sscrofa10.2 porcine assembly was used to assess the CNVs by RD. In aCGH hybridizations, the individual D4 (Duroc) was used as the reference, while the other 12 individuals as the test samples. We employed a method initially proposed by Alkan *et al*. [6] to assess the RD called CNVs with aCGH data using the individual D4 (Duroc) as the reference sample. Overall, the Pearson’s correlation coefficient between variables, defined as the log_2_(copy number-ratio) value and the mean of probe log_2_ ratios varied from 50.0% (C3) to 80.9% (R2) for each of the test animals, with an average of 62.5% (Additional file 4: Table S10). The degrees of consistency of quality assessment herein are similar with those in human and cattle [6, 15]. Additionally, we found that the level of correlation coefficient for the CNVs validation is highly dependent on the copy number differences of CNV intervals between the reference sample and the test sample, *i.e.*, the less difference of copy number, the lower the calculation of correlation coefficient. The trend of this dependence has also been clearly exemplified in Figure 4. This may be because the aCGH data is not sensitive to detect small copy number difference between test sample and reference sample due to the impact of noise signals, especially in highly duplicated regions.

In the qPCR confirmation, based on the copy numbers of every individual predicted by RD and qPCR method, we systematically assessed performance of the RD-called CNVs through three evaluation criteria in the process of validation, including the overall agreement rate of RD with qPCR results, the prediction power of RD and the positive prediction rate of RD. All the primers used and qPCR results are listed in Additional file 4: Table S11 and S12. Overall, the agreement rate, detection power and the positive prediction rate for the RD validation are 74.9%, 71.2% and 95.1%, respectively. The result demonstrated that qPCR experiments agreed well with the prediction by RD method. The discrepancies between the qPCR and results identified by RD method may be caused by potential SNPs and small indels, which influence the hybridization of the qPCR primers in some individuals, resulting in unstable quantification values or lowering primer efficiency.

Additionally, we performed qPCR validation for the CNV findings based on the original detection strategy within the same regions for comparing with those based on our improved strategy. The qPCR validation results showed that the corresponding agreement rate, detection power and the positive prediction value were 68.7%, 63.1% and 94.6%, respectively. The comparison between the two different CNV calling strategies clearly showed the credible evidences on the advantage of the improved strategy proposed herein over the original.

### Comparison with previous studies

We also compared CNVRs in this study with previous pig CNV studies [24, 25, 35, 36, 39, 41, 42]. After merging the results of recent reports, a total of 849 out of 3,131 CNVRs (27.75%) with the length of 33.02 Mb in our study overlapped with those previously reported (see Table 4). This indicates about one-third of CNVRs identified in our study was validated by previous studies, and most are firstly detected herein. Besides different algorithms for CNV calling, a difference between these NGS data-based CNV studies and the current study lies in that merely the current study employed SD information of the reference genome in the process of CNV detections, such that the short-read artifacts were removed from the detections in current study. Additionally, compared with the study by Rubin *et al*. [42], the different point is that the current study is based on individualized sequencing while that of Rubin *et al.* is based on sequencing of pooled samples. As a consequence the current study has a better power to detect CNVs with rare frequency, while the study of Rubin *et al.* is prone to find common CNVs.

**Table 4:** **Comparison between CNVRs detected in the study with those in the previous reports**

Study	CNVR detected in the previous studies							Overlaps with this study			
	Methods	Sample	CNVR	Range (kb)	Median (kb)	Mean (kb)	Total (Mb)	Count	Count percentage (%)	Total length (kb)	Length percentage (%)
Fadista et al., 2008 [35]	aCGH (385 k)	12	37	1.74-61.92	6.89	9.32	0.43	3	0.0958	21.435	0.0208
Ramayo-Caldas et al., 2010 [24]	SNP chip (60 k)	55	49	44.65-10715.82	170.96	754.59	36.97	3	0.0958	408.541	0.3974
Wang et al., 2012 [25]	SNP chip (60 k)	474	382	5.03-2702.75	142.90	250.69	95.76	61	1.9483	1845.698	1.7953
Li et al., 2012 [36]	aCGH (720 k)	12	259	2.30-1550	98.74	65.07	16.85	77	2.4593	2197.924	2.1379
Chen et al., 2012 [39]	SNP chip (60 k)	1693	565	50.39-8102.06	252.71	247.55	139.87	284	9.0706	15386.182	14.9658
Wang et al., 2013 [40]	SNP chip (60 k)	14	63	3.20 -827.21	97.85	158.37	9.98	24	0.7665	2302.633	2.2397
Rubin et al., 2012 [42]	Genome sequencing	117	1,928	0.12-175.50	3.00	5.23	10.08	305	9.7413	6777.8	6.5926
Paudel et al., 2013 [41]	Genome sequencing	16	3,118	6.00-96.00	10.00	12.74	39.72	479	15.298	16537.356	16.086
All the above		—	—	—	—	—	—	849	27.116	33018.169	32.116

Note: The comparison was based on Sscrofa 10.2 assembly (http://www.ensembl.org/Sus_scrofa/Info/Index). For CNVRs based on the other porcine assembly, we firstly converted the data to current genome coordinates using the UCSC LiftOver tool (http://genome.ucsc.edu/cgi-bin/hgLiftOver).

### Association of CNVRs with SD and other genomic features

It has been reported that CNVs may be facilitated by ancestral SDs through the occurrence of non-allelic homologous recombination (NAHR) [52], showing enrichment around ancestral SDs. To further confirm if the similar CNV formation mechanism occurs in the swine genome, we picked out SDs with <95% identity (Additional file 1: Table S3) that was postulated as the ancestral SDs that happened at earliest ~5 million years ago when *Sus scrofa* just emerged in South East Asia [45] according to the traditional sequence divergence rate of 2% per million years [53]. These putative ancestral SDs were then merged into non-overlapping regions that would be used in the enrichment analysis. Simulation results clear demonstrated the strong statistical evidence (13.9-fold enrichment; *P* < 0.001) according to the empirical distribution, indicating that the CNVRs are significantly associated with ancestral SD regions of the reference genome.

Furthermore, we also tested the correlation between CNV hotspots and ancestral SDs. Accordingly, we picked out 659 regions as CNV hotspots from 3,131 putative CNV regions (CNVRs) using the criteria that at least two of the three commercial pigs and at least two of ten Chinese pigs should be detected as having duplication/deletion within the CNVR (Additional file 4: Table S13). The simulation tests showed that 1,313 ancestral SDs overlapped with CNV hotspots while only 41 in random situation (32.0-fold; *P* < 0.001). The 32.0 fold SD enrichment for CNV hotspots was much larger than the 13.9-fold enrichment for all CNVRs, implying the special effect of ancestral SDs on evolution of CNV hotspots [52].

In addition, we explored if CNV breakpoints were enriched for GC-rich regions which were likely to show high rate of homologous recombination [54]. Based on the criteria of Berglund *et al.* [55], the breakpoints were defined by the CNVR boundaries covering a 2-kb length segment. Accordingly, we found a significantly higher GC content in these locations (44.0%; *P* < 1.0E-6) than that in the genomic background (41.6%). As reported by Berglund *et al.* [55], a GC-peak can be determined when a 500-bp sliding window centered in a 10 kb background sliding window has a 1.5-fold increase in GC content, we searched for GC-peaks across the pig genome. After performing a randomization test, we found a 1.7-fold GC-peaks enrichment in CNV breakpoints (*P* < 1.0E-6). Besides previous reports in dogs [55], the findings herein further confirmed the strong association between CNV and GC-peaks. However, the proportion of breakpoints within a 1-kb region of GC peak merely reached 3.1% in present study, which is mainly due to the sparse distribution of GC peaks across the pig genome (4.6 per Mb in average). This clues us the difference of CNV formation mechanisms among distinct species, and GC-peaks may be just one of potential CNV formation mechanisms of pig CNVs.

### Genomic effects of CNVs

To test the genomic effects of CNVs identified in the study, we compared the CNVRs identified in this study with the reported quantitative trait locus (QTL) regions collected in the pig QTL database (http://www.animalgenome.org/cgi-bin/QTLdb/SS/index, Apr 20, 2013) and human disease gene orthologs in Online Mendelian Inheritance in Man annotations (OMIM, http://omim.org/, 2013-6-19). Consequentially, some CNVRs were identified overlapping with a suite of pig QTLs (Additional file 5: Table S14) and human disease gene orthologs (Additional file 5: Table S15), providing the evidence that CNVs may be associated with or affect animal health and production traits under recent selection. Since some QTLs have too large confidence interval, we focused on the 3,789 QTLs with confidence interval less than 5 Mb. Out of the 3,789 QTLs, 1,077 (28.4%) overlapped with the CNVRs identified in this study, which are involved in a wide range of traits, such as growth, meat quality, reproduction, immune capacity and disease resistance. For the human disease gene orthologs, we found 102 CNVRs identified in the study overlapped 210 genes associated with human diseases, such as Stiff skin syndrome, Leukemia, polycythemia vera, autism, and Complement factor H deficiency. This demonstrates that, in accordance with previous studies, CNVs play an important role in phenotypic variation and are often related with disease susceptibility [9, 56].

Out of the 23,641 porcine genes locating in the 20 chromosomes, a total of 3,644 porcine genes (Additional file 6: Table S16) were completely or partially overlapped with CNVRs, including 2,773 protein-coding genes, 821 pseudo genes, 3 tRNA genes, 17 miscRNA genes and 30 genes with other types. It is notable that these genes are distributed merely in 1,820 CNVRs (58.1%) of all identified CNVRs, *i.e.*, the remaining 41.9% CNVRs do not contain any annotated genes. The distribution of genes among CNVRs from the present studies is similar with those in other studies [4, 15, 25]. To test if the genes are enriched in these CNVRs, an empirical distribution of genes among CNVRs were constructed through 10,000 simulations. Consequentially, we found that the genes trended to enrich within the CNVRs (1.8-fold enrichment; *P* < 0.001), especially for the protein-coding genes (1.6-fold enrichment; *P* < 0.001), reflecting that porcine CNVs occurred in gene-rich regions in the genome.

In order to provide insight into the functional enrichment of the CNVs, Gene Ontology (GO) and Kyoto Encyclopedia of Genes and Genomes (KEGG) pathway analyses were performed for the genes in CNVRs with the DAVID bioinformatics resources. The GO and pathway analyses revealed that there were 12 significant terms (Additional file 6: Table S18) and 8 significant pathways after Benjamini correction. Our results are consistent with previous studies in other mammals that CNVRs are particularly enriched in genes related to immunity, sensory perception of the environment (*e.g.* smell, sight, taste), response to external stimuli and neurodevelopmental processes [57].

### Copy number variable genes in the CNVRs

According to the copy windows, we estimated the CNs for all genes in the CNVRs identified by RD. In total, there were 2,223 genes assigned copy numbers (Additional file 6: Table S16). The results showed that some of genes with high copy numbers belong to some multiple-member gene families, such as olfactory receptor (*OR*), protein *FAM22G*, UDP-glucuronosyltransferase, ATP-binding cassette subfamily G, butyrophilin subfamily 1 member A1, leukocyte immunoglobulin-like receptor subfamily, melanoma-associated antigen, tumor necrosis factor receptor superfamily member, and cytochrome P450. This is consistent with previous studies that high copy number genes often belong to multiple-member gene families [5, 15].

Excepting the above mentioned copy number variable gene families and those uncharacterized genes, there were 123 protein-coding genes with copy number range more than 2.0 among the individuals investigated (Additional file 6: Table S19). Further probing the potential functions of these 123 copy number variable genes, we found a suite of genes related to the immune response, meat quality, sexual and reproduction ability, nutrients metabolism and coat color, which representing a valuable resource for future studies on the relation between CNV genes and phenotype variation.

In particular, the *KIT* gene is the most obvious copy number variable gene with functional significance, which has been confirmed that gene duplication and a splice mutation leading the skipping of exon 17 is responsible for the dominant white phenotype [58, 59]. In our studies, we estimated the copy numbers of the *KIT*, and obtained the copy number of the *KIT* gene of 4.50 and 3.81 in the solid white breeds Yorkshire and Landrace, respectively, while about two copies (ranged from 1.71 to 1.97) in all other pigs having colored phenotypes (see Additional file 2: Figure S5 for read depth of all samples within the region). This is consistent with the causative relation between *KIT* duplication and dominant white coat color identified before [58, 59]. In particular, no CNVs were found in the *KIT* gene of the Rongchang pig (copy number = 1.94), which is the Chinese indigenous breed that is characterized for its solid white coat color on body and some black patches around the eyes and ears. The result confirmed the previous finding that the white coat colors in Chinese pigs were not caused by the dominant white allele of *KIT* [60].

**Figure 5:**
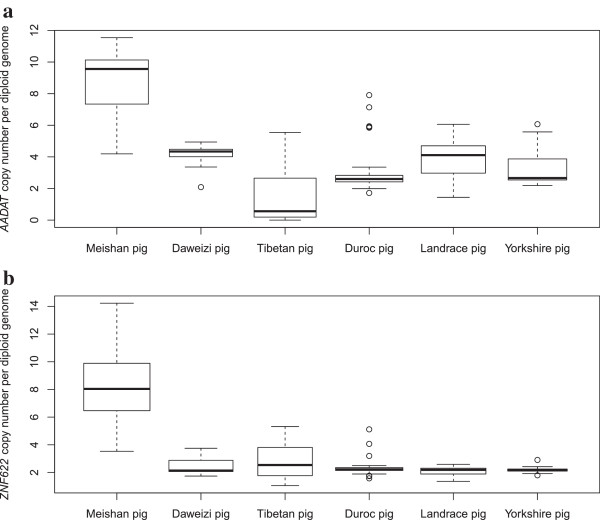
**Box plot of gene copy number quantification for** ***AADAT*** **(a) and** ***ZNF622*** **(b).** The gene copy number was measured by qPCR assays across six pig breeds, including Meishan pig, Daweizi pig, Tibetan pig, Duroc pig, Landrace pig and Yorkshire pig. Boxes indicate the interquartile range between the first and third quartiles, and the bold line indicates the median. Whiskers represent the minimum and maximum within 1.5 times the interquartile range from the first and third quartiles. Outliers outside the whiskers are shown as circles.

Among these 123 copy number variable genes, some genes were existed in specific breed or population. For instance, kynurenine/alpha-aminoadipate aminotransferase (*AADAT)* and zinc finger protein 622 (*ZNF622)* have extremely high copy numbers in the re-sequenced Meishan individuals (above 5.0 and 9.0 for *AADAT* and *ZNF622*, respectively) compared to the other individuals. To further explore copy number distributions of them at population levels across multiple breeds and mine potential function contributing to formation of particular breed features, we determined the absolute copy numbers of these two genes via qPCR. A total of 174 unrelated individuals from six pig breeds (Meishan, Tibetan, Daweizi, Yorkshire, Landrace and Duroc) were employed in the confirmation study. The primers used, average copy number estimates for these two genes in each breed are presented in Figure 5 and Additional file 6: Table S20. The validation outcomes showed the consistent tendency with that in RD analyses, *i.e.*, both *AADAT* and *ZNF622* have above 8.0 in average in Meishan breeds, being approximately 2- to 4- folds higher than those in the other five breeds. In mouse, the activity of the rat and mouse’s *AADAT* gene is associated with the transamination of alpha-aminoadipic acid, which is the final step in the major pathway (the saccharopine pathway) for the catabolism of L-lysine (*AADAT* NCBI reference). *ZNF622* pertains to the zinc finger gene family and has been proved involved in embryonic development [61]. Concerning potential function of *AADAT* and *ZNF622*, we can speculate that extraordinary high copy numbers of *AADAT* and *ZNF622* likely account for the typical features, such as high fertility, roughage-resistance, lower growth rate in Meishan pigs.

## Discussion

In current study, we developed a SD map of reference genome with 2,860 intervals and systemically performed the first genome-wide analysis of recent SDs using the newest build of porcine genome (*Sscrofa* 10.2) by both WGAC and WSSD methods. The construction of SD map herein presented essential SD features of pig genome, like inter-/intra-chromosomal patterns of SDs and the identity of pairwise alignments, *etc.*, aiding understanding of genome innovation, genomic rearrangements, and occurrences of CNV hotspots within species [4, 18, 51, 62]. It has been reported [52, 63] that SDs may contribute to the formation of some CNVs through the occurrence of NAHR mechanisms. Certain ancestral SDs that were transmitted to their descendants may facilitate separate NAHR in them, leading to the genesis and maintenance of CNVs. The impact of SD on the CNVs has also been reflected by our findings that there are significant association between the ancestral SDs and CNVRs and CNV hotspots. From the practical perspective, the reference genome SD database generated in our study also provides a very useful calibration for filtering short-read artifacts, which is necessary for duplication/deletion detection in WSSD analyses of individual NGS data. Besides the SD map of the pig reference genome, we also constructed a CNV picture involving 3,131 unique regions using WSSD through re-sequencing 13 highly representative individuals from ten distinct breeds or populations. To our knowledge, this is the highest resolution CNV map so far in the pig genome. The abundance of CNV outcomes in our study further confirmed our initial expectation that individuals from multiple breeds, especially Chinese indigenous breeds, can greatly contribute to the CNV identification. The alteration of copy numbers of these genes within CNVRs may be responsible for the genetic diversity among diverse breeds with distinctive natures, especially for those entailed in various Chinese indigenous breeds.

Additionally, we further confirmed the previous findings that the duplication of *KIT* gene is responsible for the dominant white phenotypic breeds like Landrace and Yorkshire, while with the exception of Chinese indigenous solid white breeds like Rongchang pig surveyed. In our study, besides those multiple-member gene families and uncharacterized genes, a total number of 123 copy number variable genes have been mined within CNVRs across 13 individuals with different genetic backgrounds from ten distinct breeds, which merit functional validation in depth in follow-up studies. Especially, the two genes, *AADAT* and *ZNF622*, entail obviously high copy numbers merely in Meishan pigs, which can be considered as promising candidate functional genes in CNV-related association studies in the future.

In CNV detection, we adopted the read depth specific analytical tool mrsFAST to map sequence reads to the reference genome. Compared with other read depth methods considering merely one mapping location per read, mrsFAST can map sequence reads to all possible locations for a sequence read, demonstrating advantages of detection power in searching for SD regions. Highlights in our analyses involve three aspects:

Firstly, we proposed an enhanced strategy to determine three different types of sliding windows to adjust the bias in CNV calling due to fragmented sequences in the process of hard masking of the reference genome, especially for NGS data with long sequence reads. We defined sliding windows based on unique hits where short-reads can be forward aligned with the reference sequence rather than non-masked bases employed in the original mrCaNaVaR. This could largely conquer the inaccuracy of read depth calculation for each type of sliding windows arising from hard masking of the reference genome. Accordingly, we could use more reliable read depth statistics to infer duplication/deletion and estimate copy number, leading to better sensitivity and specificity of duplication/deletion detection as well as increased accuracy of copy number estimation. The performance gain of the enhanced strategy over the original has been verified by qPCR as well as through simulation analyses.

Secondly, we probed formation signatures of both SDs of the pig reference genome and individualized CNVs in an integrated fashion. Based on the identified CNVs and SDs, we systemically explored associations of CNVRs with various genome features, building a comprehensive profile of genome-wide CNVs in swine.

Finally, we exploited CNVs across the pig genome among ten distinct breed populations and dug out corresponding genes within these specific regions, which may be considered as the most important copy number variable genes responsible for genetic diversity and specific breed features. Furthermore, we predicted absolute copy number of completely all genes within CNVRs across the genome and sifted out 123 protein-coding genes. Most of these specific CNVs and CNV-related genes are firstly reported by our studies.

The WGAC and WSSD methods employed in this study have demonstrated obvious advantages. However, some limitations still exist in detecting SDs and CNVs. Specifically, WGAC can identify whole-genome SDs with the length of >1 kb and determine accurate SD breakpoints, but it does depend on the whole genome assembly of the individual investigated. It is also difficult for WGAC to dissect high-identity SDs, which should be further filtered by WSSD. The WSSD method has inevitable weakness in determining breakpoint due to its nature of relying on pre-defined sliding windows. Considering the sliding length (generally set as 1 kb), the WSSD method can merely identify a rough position of CNV breakpoint.

The inaccuracy of CNV breakpoint determination limited our view about the CNV formation. In this study we specially focused on recurrent CNVs instead of non-recurrent ones. Recurrent CNVs show recurrent breakpoints in SDs, arising by meiotic unequal or non-allelic homologous recombination [64]. In contrast, non-recurrent CNVs have unique breakpoints that are not dependent on SDs, possibly arising by nonhomologous end-joining (NHEJ), microhomology-mediated end-joining (MMEJ), fork stalling and template switching (FoSTeS), or microhomology-mediated break-induced replication (MMBIR) [64]. Our study showed a significant association between CNVs and ancestral SDs in pig genome, giving evidence on the abundance of recurrent CNVs in our results. Though it is possible to distinguish recurrent and non-recurrent CNVs based on their differences in breakpoint distribution (common versus variable) and association with SDs (dependent versus independent) [64], the ambiguity of CNV breakpoints due to the shortness of the WSSD method made it unfeasible to achieve this goal.

## Conclusion

In the present study, we proposed an enhanced strategy to determine three different types of sliding windows to adjust the bias in CNV calling due to fragmented sequences in the process of hard masking of the reference genome, and then exploited both segmental duplications (SDs) and individualized CNVs across the pig genome among ten distinct breed populations and dug out corresponding genes within these specific regions. Our studies lay out one way for characterization of CNVs in the pig genome, provide insight into the pig genome variation and prompt CNV mechanisms studies when using pigs as biomedical models for human diseases.

## Methods

### Ethics statement

The whole procedure for collection of the ear tissue samples of all animals was carried out in strict accordance with the protocol approved by the Institutional Animal Care and Use Committee (IACUC) of China Agricultural University.

### Selection of pig breeds and experimental animals

In this study, a total number of 13 pig samples originated from ten distinct populations were chosen for sequencing. These samples comprised one Asian wild pig, three modern commercial pigs (1-Landrace, 1-Duroc and 1-Yorkshire), and nine pigs selected from six Chinese indigenous breeds (2-Tibetan pig, 2-Diannan small-ear pig, 2-Meishan pig, 1-Min pig, 1-Daweizi pig, and 1-Rongchang pig). Duroc, Yorkshire and Landrace are considered as the representatives of modern commercial breeds, while the six Chinese indigenous breeds, each belonging to a specific population type, are considered as the representatives of Chinese indigenous population. The illustration of the features of six Chinese indigenous breeds were detailed elsewhere [65]. Furthermore, to explore the phylogeny relationships among them, the 13 individuals were genotyped by Porcine SNP60 BeadChip (Illumina). SNPs with 100% call rate (*n* = 55,438) from these 13 samples were used to construct the Neighbor-joining tree using MEGA version 5.0 [66]. As shown in Additional file 2: Figure S6, the experimental samples can well represent diverse populations of the commercial breeds and Chinese indigenous breeds.

### Re-sequencing and data acquisition

Genomic DNA of 13 individuals was extracted from the ear tissue using Qiagen DNeasy Tissue kit (Qiagen, Germany). All DNA samples were analyzed by spectrophotometry and agarose gel electrophoresis and sequenced using the Illumina HiSeq 2000 technology. All paired-end reads reached the length of 100 bp, with an average insert size of 460–490 bp and the standard deviation of 11–14 bp estimated for all samples. The reads which contain more than 50% low quality bases (quality value ≤5) or more than 10% N bases were removed. The Q20 bases rate of reads of each individual is above 90%.

For the sequenced Duroc sow 2–14, we downloaded its draft genome sequence (*i.e. Sus scrofa* 10.2 reference assembly) from ftp://ftp.ensembl.org/pub/release-67/fasta/sus_scrofa/dna/ and corresponding NGS data from DDBJ (ftp://ftp.ddbj.nig.ac.jp/ddbj_database/dra/fastq/ERA009/ERA009086/) for the use of sequence alignment and SD map construction.

### Developing an enhanced strategy in WSSD analyses

**Figure 6:**
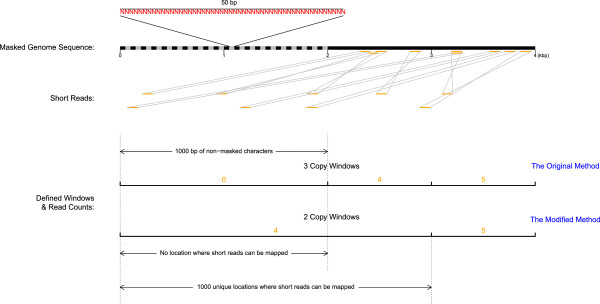
**Illustration of the modified method of windows definition.** As showed in the top of the graph, on a 4 kb genome sequence, black regions represent A/T/C/G characters and grey regions denote N characters. Due to hard masking, 50 bp N blocks are uniformly distributed on the first 2 kb sequence, resulting in no any 100 bp reads being mapped there. According to copy window definition by the original method that every 1,000 bp of non-masked characters are defined as one copy window, the whole 4 kb long masked genome sequence is divided into three copy windows and the first 2 kb long sequence is defined as one copy window. The three copy windows have read counts of 0, 4 and 5, respectively. Thus the hard masked sequence of the first 2 kb may be considered as deletion. In contrast, the modified method we proposed herein defines every 1,000 unique locations where short reads can be mapped as one copy window, so the masked genome sequence is accordingly divided into two copy windows with read counts of 4 and 5, respectively, avoiding false prediction of deletion for the hard masked region.

In RD approach for WSSD analyses, hard masking of genome sequences is a routine process for generating more accurate read depth statistics of long window, short window and copy window. However, hard masking may produce biases in both duplication and deletion detection, especially for long sequence reads (*e.g.*, ≥100 bp). We define here this kind of bias as the fragmentation effect, which received seldom attention preciously since it does not matter due to the length of reads is merely 36 bp in most of earlier studies [5, 6, 15]. To reduce potential fragmentation effects, we modified mrCaNaVaR to optimize the way in defining the three windows, i.e., long window, short window and copy window. Specifically, the sizes of windows are based on the number of unique hits where short-reads can be forward aligned with the reference sequence rather than the accumulative counts of non-masked characters employed in the original mrCaNaVaR. Accordingly, the biases in duplication/deletion detection and CN estimation due to fragmentation effects can be largely corrected. A more intuitive illustration on the so-called fragmentation effect and our improved strategy were also given in Figure 6. The more details on our enhanced strategy were given in the supplementary method, Section 1 of Additional file 7. To further validate the performance of the enhanced strategy herein, extensive simulation analyses were conducted to systematically compare the detection power, accuracy of copy number estimates between the original and the enhanced strategy herein (for details, see Additional file 7, Section 2).

### Construction of SD map for the reference genome

We performed both WGAC and WSSD analyses to map SDs based on *Sus scrofa* 10.2 genome assembly (*Sscrofa* 10.2). These two analytical algorithms were initially performed in human genome [7, 49], which can provide comprehensive and complementary SD findings with different levels of sequence identity and resolution. The specific process for porcine SD map development by WGAC and WSSD approaches is detailed in the supplementary method, Section 1 of Additional file 7.

After finishing both WGAC and WSSD analyses for the reference genome, to further remove artifactual duplications, we filtered the WGAC alignments of ≥94% identity with the WSSD dataset following the criteria proposed by [7]. We finally developed a pig SD database based on the union of low-identity WGAC (<94%), filtered high-identity WGAC (≥94%) and the WSSD estimates.

### Detection of duplication/deletion for re-sequenced individuals

Based on the SD findings in the pig reference genome, we employed RD method to detect both SDs/deletions for the re-sequenced samples through running mrsFAST and our improved mrCaNaVaR program. The specific steps for SDs/deletions calling are given in the supplementary method, Section 1 of Additional file 7.

### Validation of pig CNVs using aCGH and qPCR

We employed aCGH with a custom-designed 2.1 M oligonucleotide array (Roche-NimbleGen) based on the *Sscrofa*10.2 porcine assembly for CNV validation. The array contained 2,167,769 oligonucleotide probes (50–75 mers), with an average interval of 889 bp between probes, covering 18 autosomes and two sex chromosomes. Details for aCGH analyses are presented in the supplementary method, Section 1 of Additional file 7, Section 1.

Besides aCGH, qPCR was used to validate CNVRs identified by NGS data in the study. The control region is determined within the region of the glucagon gene (GCG), which is highly conserved between species and has been proved to have a single copy in animals [67]. The specific process of qPCR analyses and the criteria for quantifying the performance of RD-based CNV calling are detailed in the supplementary method, Section 1 of Additional file 7.

### Gene content and functional analyses

Pig CNVRs were annotated using NCBI gene information (ftp://ftp.ncbi.nih.gov/genomes/Sus_scrofa/mapview/seq_gene.md.gz; ftp://ftp.ncbi.nlm.nih.gov/gene/DATA/GENE_INFO/Mammalia/Sus_scrofa.gene_info.gz). Those genes overlapping with CNVRs completely or partially were considered as copy number variable and picked out for further analyses. Copy number of each variable gene was estimated as the median of copy numbers corresponding to copy windows within the region of the gene. To provide insight into the functional enrichment of copy number variable genes, annotation analyses were performed with the DAVID (http://david.abcc.ncifcrf.gov/) for Gene Ontology (GO) terms and Kyoto Encyclopedia of Genes and Genomes (KEGG) pathway analyses. Since only a limited number of genes in the pig genome have been annotated, we firstly converted the pig EntrezGene IDs to orthologous human RefSeq genes by BioMart (http://www.biomart.org/) ahead of GO and pathway analyses. Statistical significance was assessed using a modified Fisher’s exact test while considering multiple testing correction based on Benjamini’s method.

### Pig CNV distribution and association with SDs and other genomic features

We performed simulations to probe if the identified CNVs are associated with SD regions and other genomic features, such as protein-coding genes (ftp://ftp.ncbi.nih.gov/genomes/Sus_scrofa/mapview/seq_gene.md.gz). Specifically, for SD region association analyses, we randomly assigned each of identified CNVRs a putative position with no overlap with each other in the genome. The number of SDs overlapping with CNVRs was calculated in each simulation, and finally we created empirical distribution of the hits via 10,000 independent replications. Thus the significance of pig CNV enrichment/depletion in SD regions could be determined by the thresholds based on the empirical distribution. Similarly the association analyses were further conducted for other genomic features investigated, i.e., genes and protein-coding genes.

### Data access

The complete SNP array data and aCGH data have been submitted to the Gene Expression Omnibus (http://www.ncbi.nlm.nih.gov/geo/) and released under the accession number GSE46733 and GSE46847, respectively.

## Electronic supplementary material

Additional file 1: **Description: The file contains Supplemental Tables S1 to S4.** (XLSX 2 MB)

Additional file 2: **Description: The file contains Supplemental Figures S1 to S6.** (PDF 1 MB)

Additional file 3: **Description: The file contains Supplemental Tables S5 to S9.** (XLSX 1 MB)

Additional file 4: **Description: The file contains Supplemental Tables S10 to S13.** (XLSX 129 KB)

Additional file 5: **Description: The file contains Supplemental Tables S14 to S15.** (XLSX 230 KB)

Additional file 6: **Description: The file contains Supplemental Tables S16 to S20.** (XLSX 2 MB)

Additional file 7: **Description: The file contains supplementary methods.** (DOC 206 KB)
