# Supplementary material for: Global copy number analyses by next generation sequencing provide insight into pig genome variation
Source: BMC Genomics. 2014 Jul 14;15(1):593. doi: 10.1186/1471-2164-15-593 (PMC4111851; doi:10.1186/1471-2164-15-593)
Supplement: Supplementary file 2 — Additional file 2: Description: The file contains Supplemental Figures S1 to S6. (PDF 1 MB) [file 12864_2014_6278_MOESM2_ESM.pdf]

Supplemental figure legends

Figure S1. Distribution of WGAC pairwise alignments with identity <99%

Figure S2. Distribution of unplaced scaffolds against the proportion of duplicated bases

Figure S3. SD contents of different chromosomes for pig reference genome

Figure S4. A composite map of CNVs, CNVRs, CNV hotspots and SDs based on the *Sus scrofa* 10.2 genome assembly
A1, wild boar; C3, Landrace; D4, Duroc; DN1 and DN5, Diannan small-ear pig; M2, Min pig; MS7 and MS8, Meishan pig; R2, Rongchang pig; W1, Daweizi pig; Y2, Yorkshire; Z2 and Z5, Tibetan pig.

Figure S5. Read depth map around *KIT*
For each individual, the horizontal line indicates the averaged read depth of whole-genome 2-copy copy windows. Read depth of copy windows was painted in different colors according to their values: green for average-2×STDEV<RD<average+2×STDEV, red for RD ⩾ average+3×STDEV, blue for RD⩽average-3×STDEV, and grey for other RD values. Tracks on the bottom of the figure include human Ensembl proteins, genes, contigs and %GC (showed from top to bottom), sourcing from Ensembl. Ref, the Duroc sow 2-14; A1, wild boar; C3, Landrace; D4, Duroc; DN1 and DN5, Diannan small-ear pig; M2, Min pig; MS7 and MS8, Meishan pig; R2, Rongchang pig; W1, Daweizi pig; Y2, Yorkshire; Z2 and Z5, Tibetan pig.

Figure S6. Dendrogram of 13 individual pigs from 10 distinct breeds using high density SNP data
The values at the nodes are the percentages of bootstrap results from 1,000 replications of re-samplings, which indicate the reliability of bootstrap test. A1, wild boar; C3, Landrace; D4, Duroc; DN1 and DN5, Diannan small-ear pig; M2, Min pig; MS7 and MS8, Meishan pig; R2, Rongchang pig; W1, Daweizi pig; Y2, Yorkshire; Z2 and Z5, Tibetan pig.

Figure S1

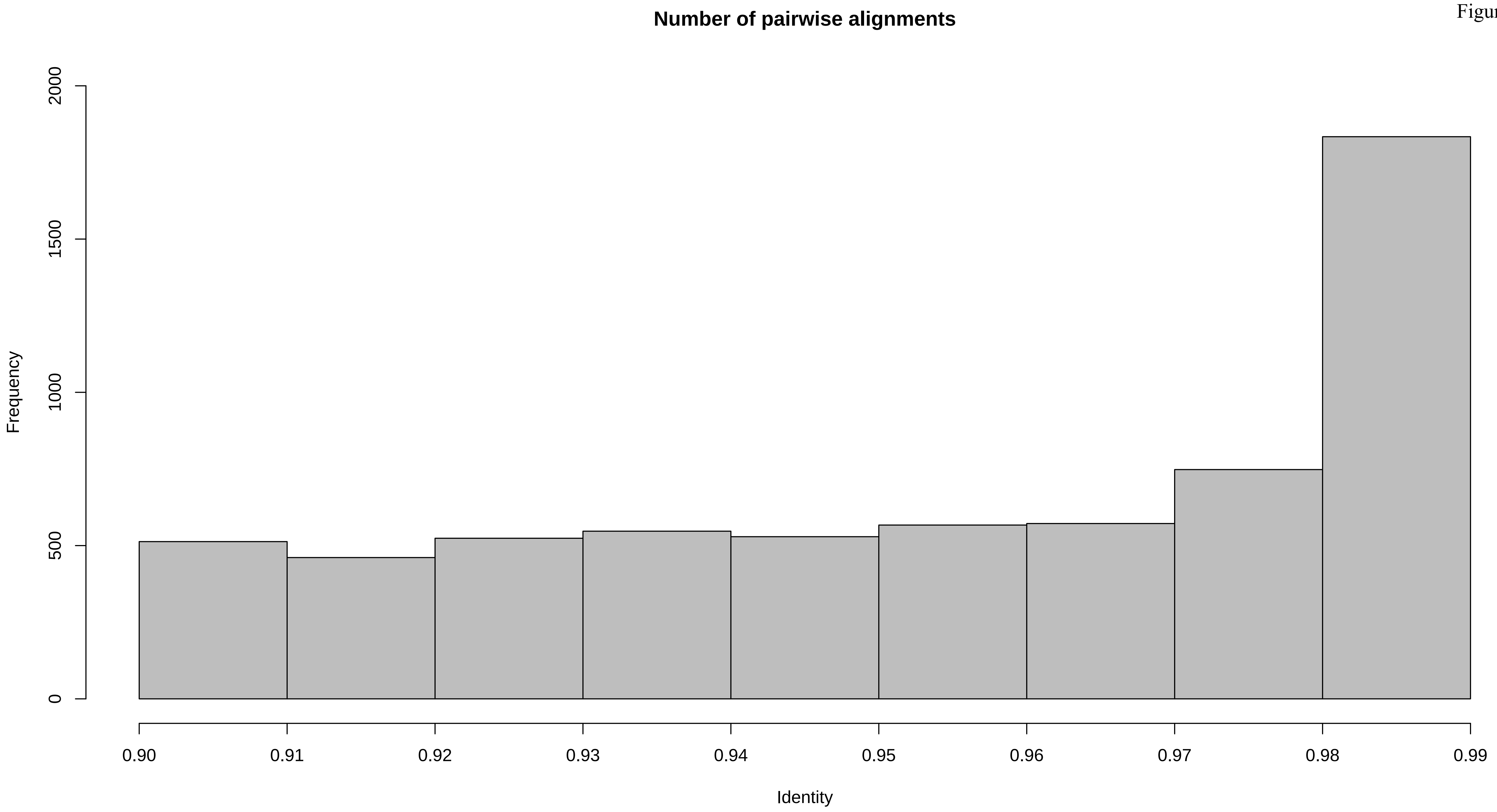

Number of pairwise alignments
Frequency
0
500
1000
1500
2000
0.90
0.91
0.92
0.93
0.94
0.95
0.96
0.97
0.98
0.99
Identity

Figure S2

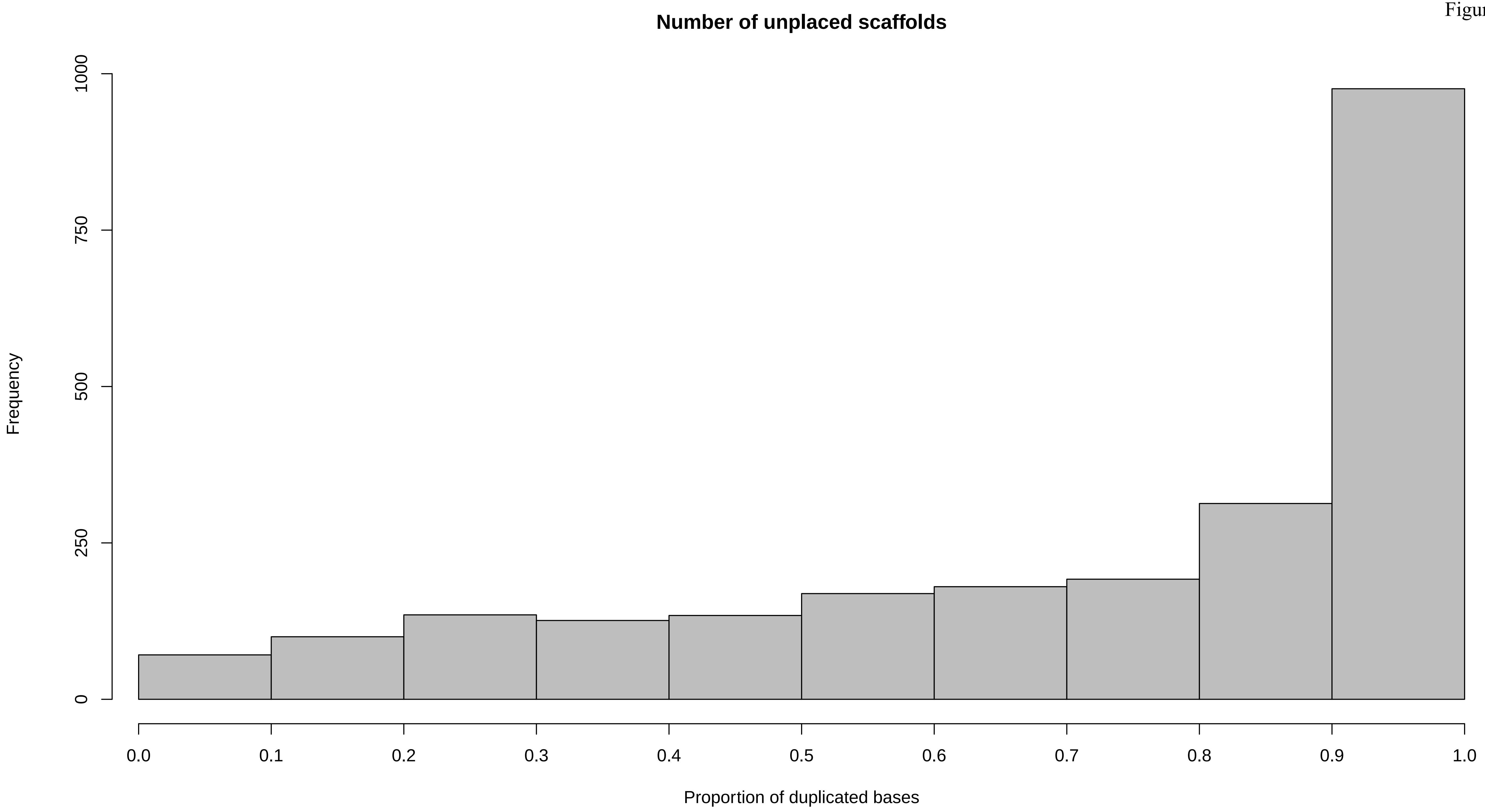
Number of unplaced scaffolds
Frequency
0
250
500
750
1000
0.0
0.1
0.2
0.3
0.4
0.5
0.6
0.7
0.8
0.9
1.0
Proportion of duplicated bases

Figure S3

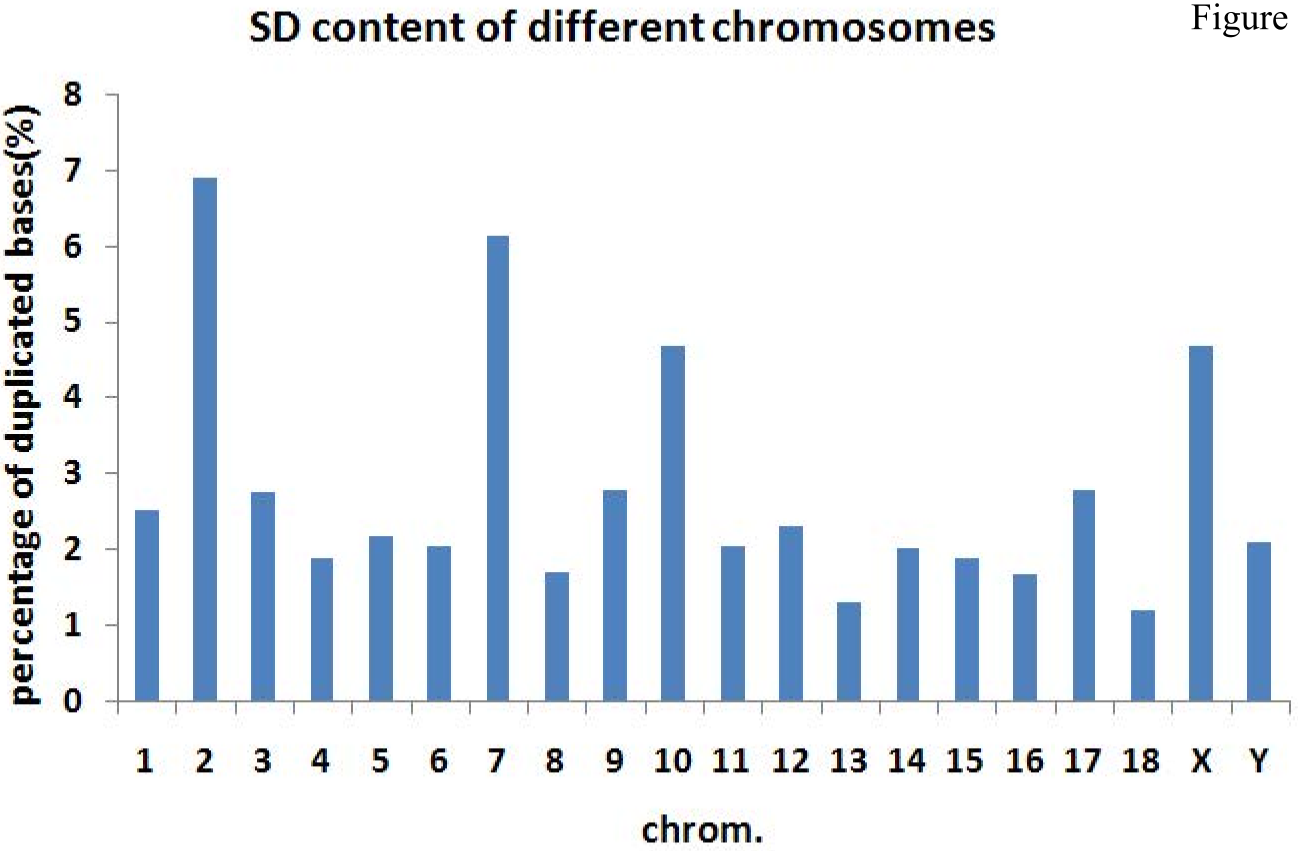
SD content of different chromosomes
percentage of duplicated bases(%)
0
1
2
3
4
5
6
7
8
1
2
3
4
5
6
7
8
9
10
11
12
13
14
15
16
17
18
X
Y
chrom.

Figure S4_chr1

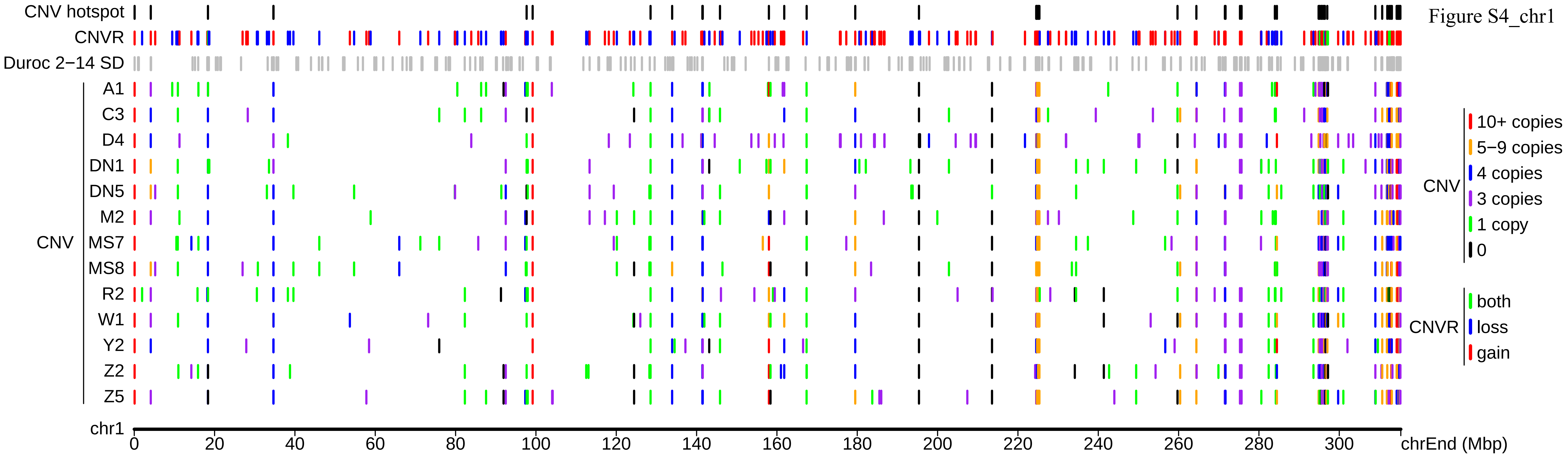

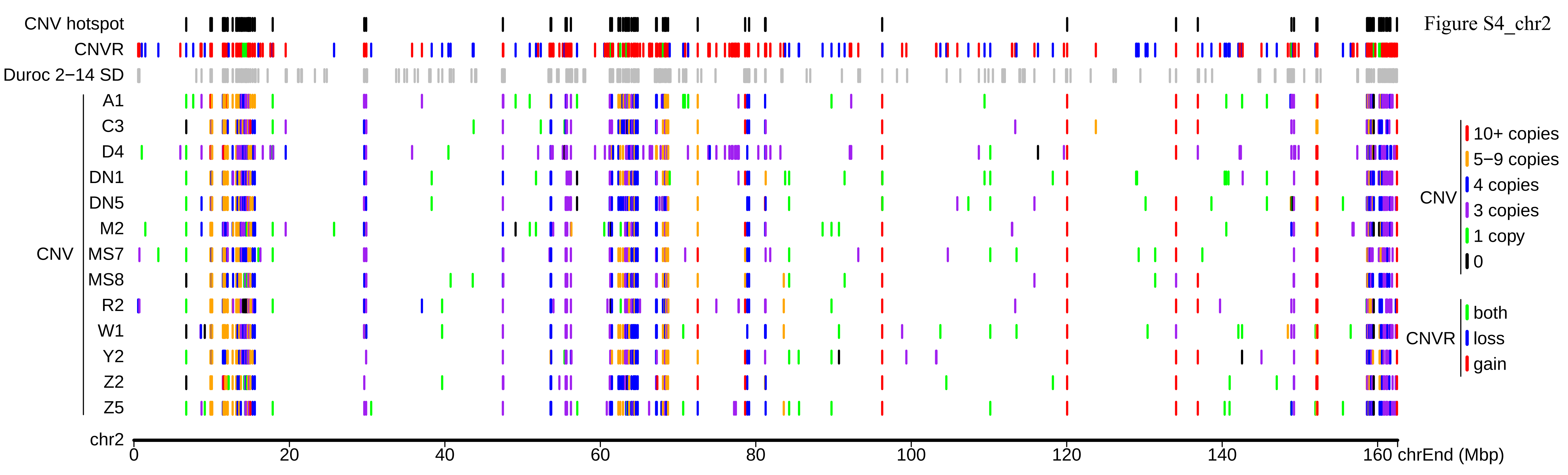

Figure S4_chr2
CNV hotspot
CNVR
Duroc 2−14 SD
CNV
A1
C3
D4
DN1
DN5
M2
MS7
MS8
R2
W1
Y2
Z2
Z5
chr2
0
20
40
60
80
100
120
140
160
chrEnd (Mbp)
CNV
10+ copies
5−9 copies
4 copies
3 copies
1 copy
0
CNVR
both
loss
gain

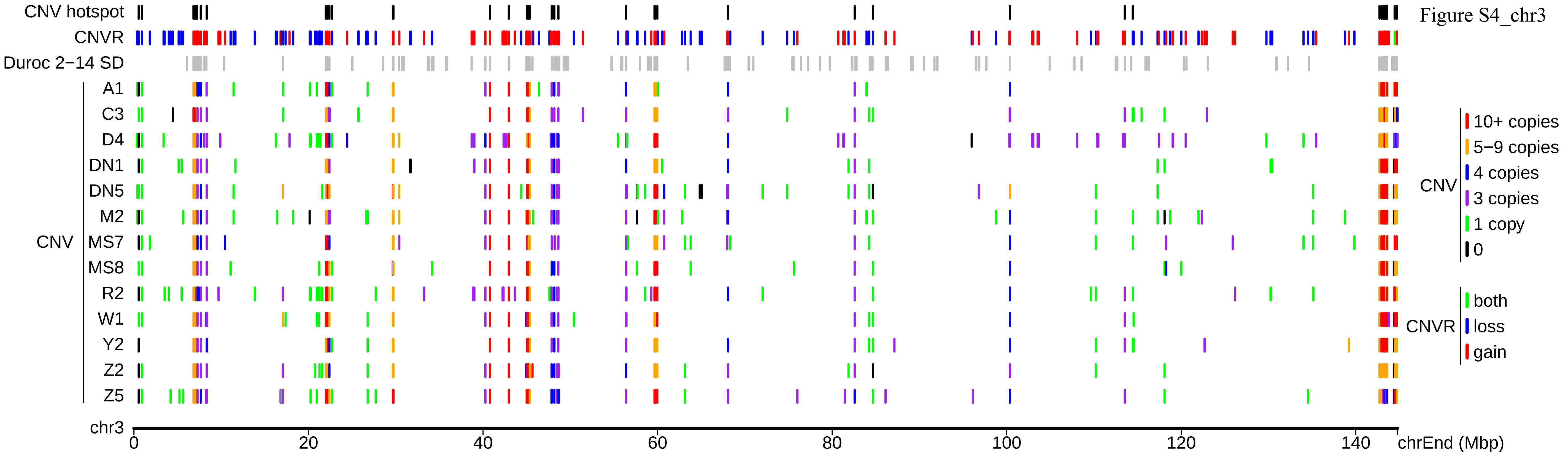
Figure S4_chr3
CNV hotspot
CNVR
Duroc 2−14 SD
CNV
A1
C3
D4
DN1
DN5
M2
MS7
MS8
R2
W1
Y2
Z2
Z5
chr3
0
20
40
60
80
100
120
140
chrEnd (Mbp)
CNV
10+ copies
5−9 copies
4 copies
3 copies
1 copy
0
CNVR
both
loss
gain

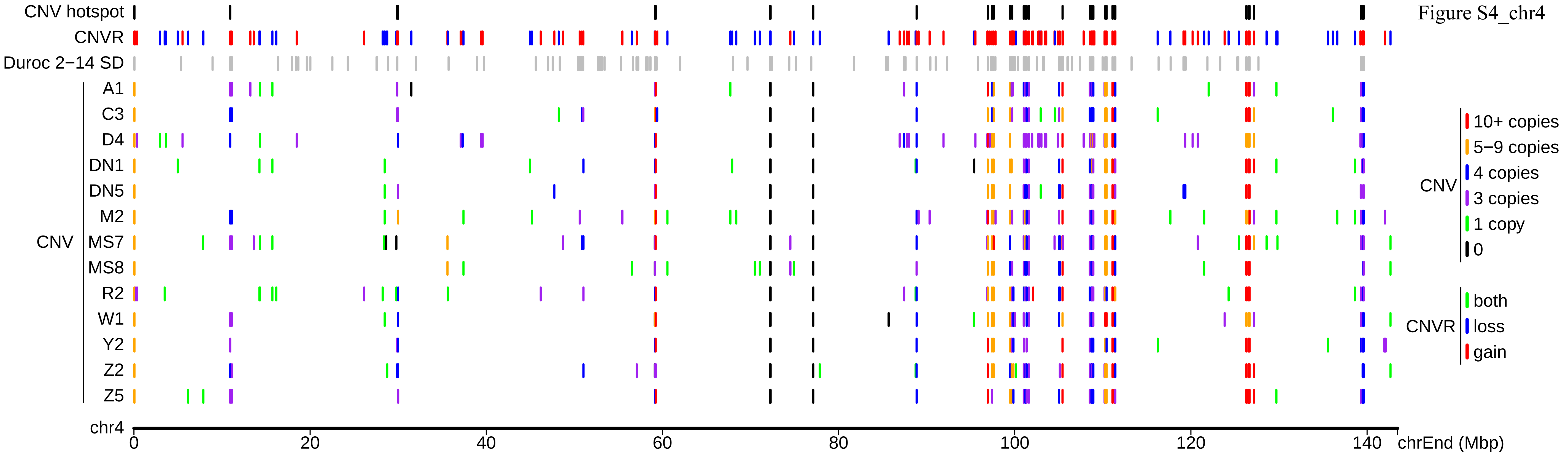
Figure S4_chr4
CNV hotspot
CNVR
Duroc 2−14 SD
CNV
A1
C3
D4
DN1
DN5
M2
MS7
MS8
R2
W1
Y2
Z2
Z5
chr4
0
20
40
60
80
100
120
140
chrEnd (Mbp)
CNV
10+ copies
5−9 copies
4 copies
3 copies
1 copy
0
CNVR
both
loss
gain

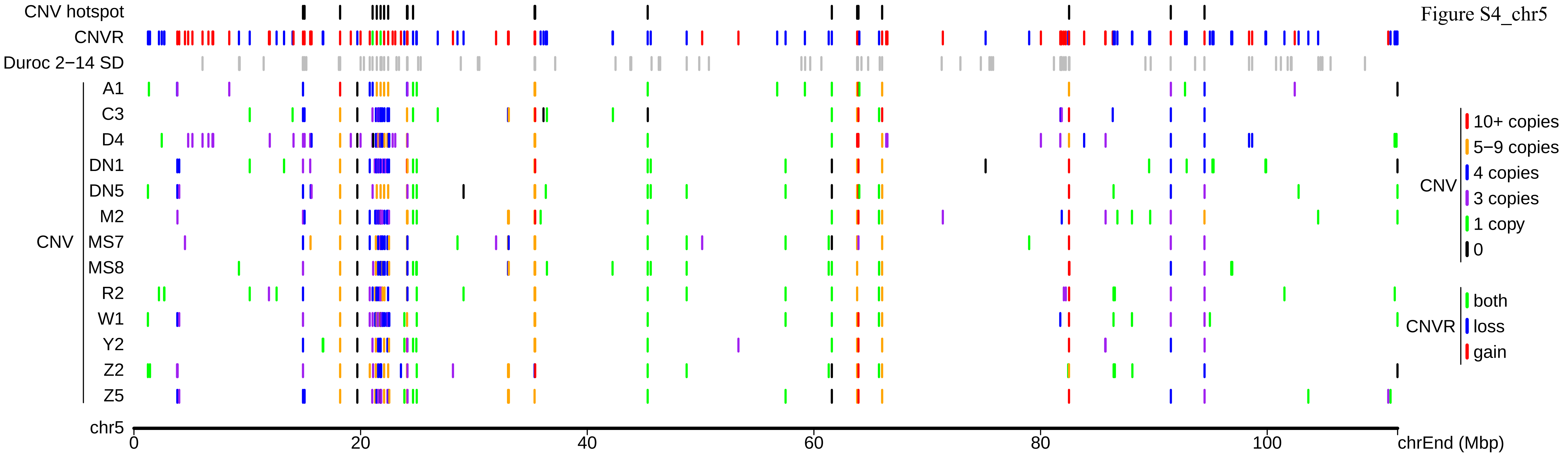
Figure S4_chr5
CNV hotspot
CNVR
Duroc 2−14 SD
CNV
A1
C3
D4
DN1
DN5
M2
MS7
MS8
R2
W1
Y2
Z2
Z5
chr5
0
20
40
60
80
100
chrEnd (Mbp)
CNV
10+ copies
5−9 copies
4 copies
3 copies
1 copy
0
CNVR
both
loss
gain

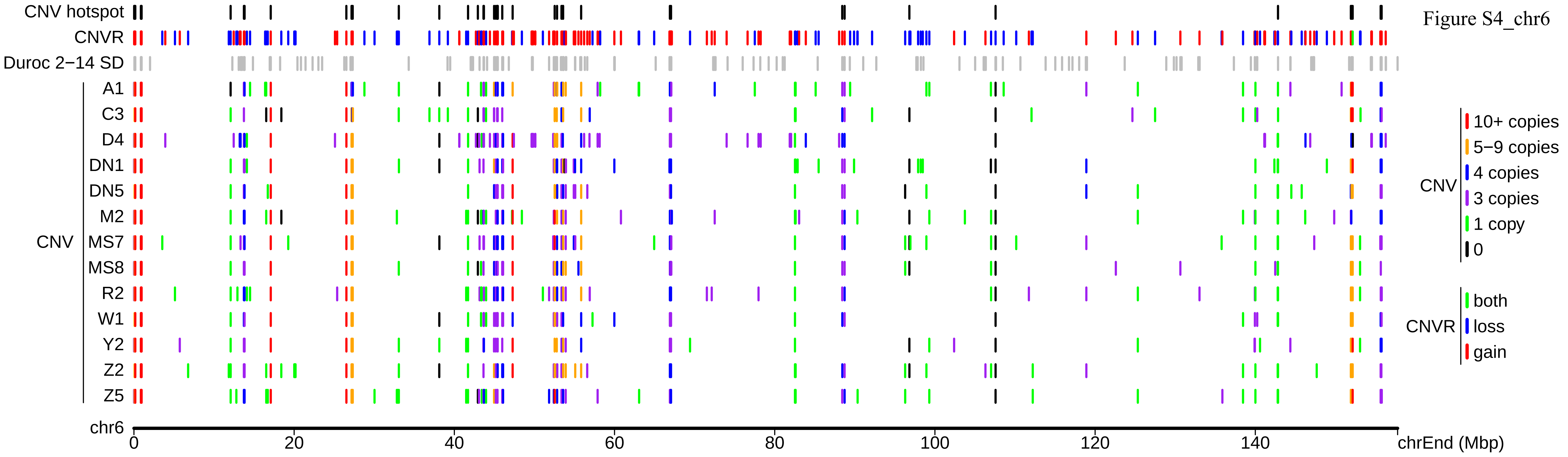

Figure S4_chr6
CNV hotspot
CNVR
Duroc 2−14 SD
CNV
A1
C3
D4
DN1
DN5
M2
MS7
MS8
R2
W1
Y2
Z2
Z5
chr6
0
20
40
60
80
100
120
140
chrEnd (Mbp)
CNV
10+ copies
5−9 copies
4 copies
3 copies
1 copy
0
CNVR
both
loss
gain

Figure S4_chr7

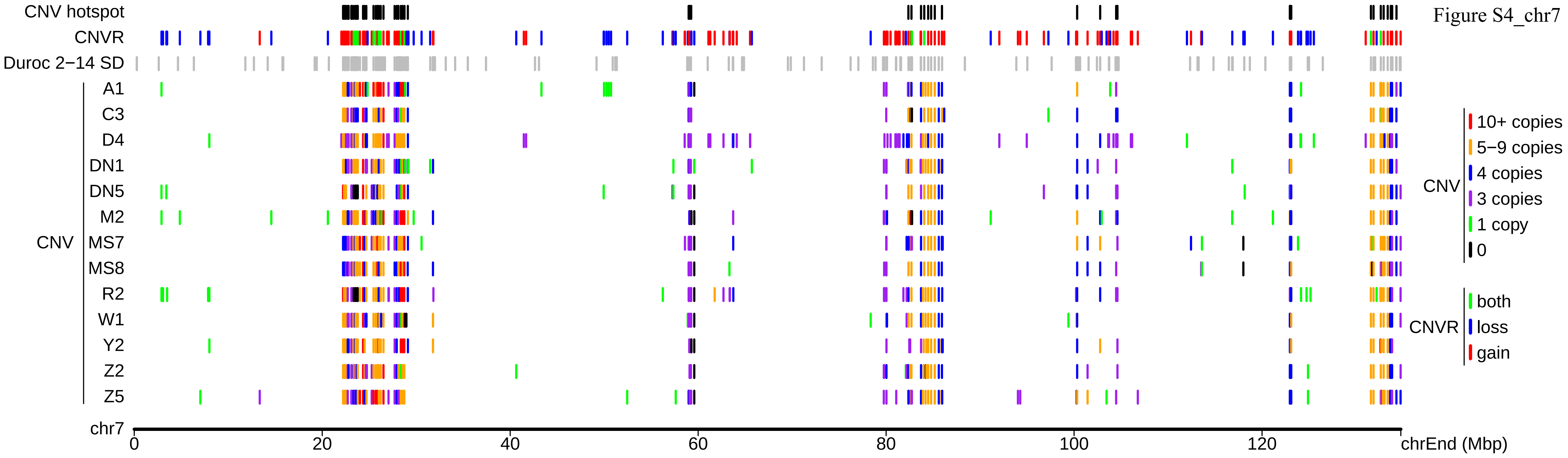

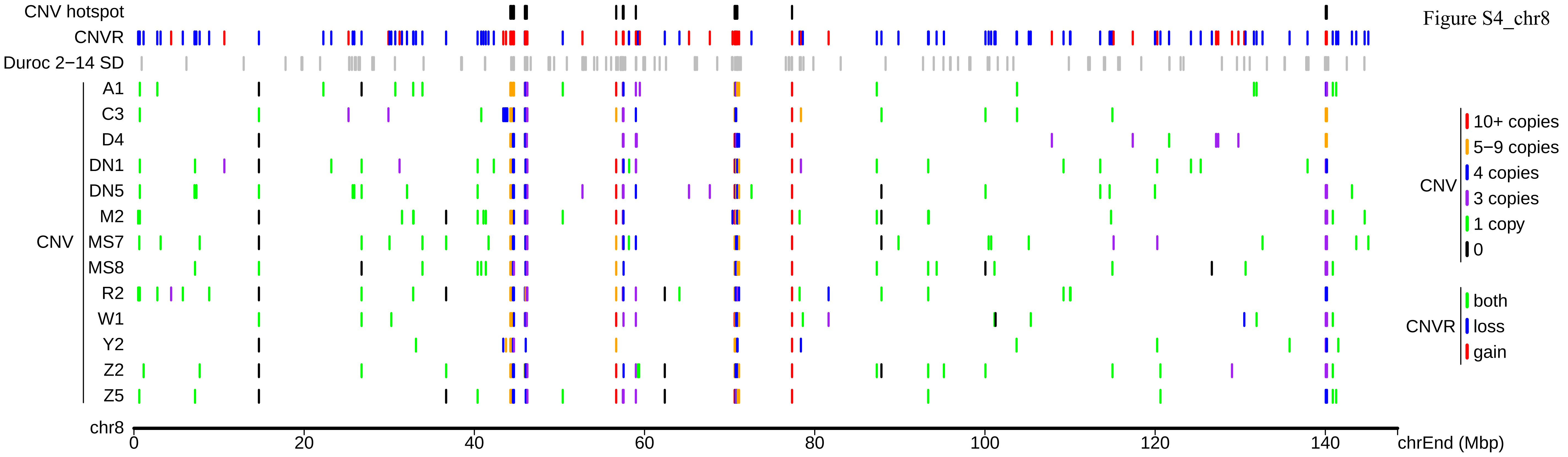
Figure S4_chr8
CNV hotspot
CNVR
Duroc 2−14 SD
CNV
A1
C3
D4
DN1
DN5
M2
MS7
MS8
R2
W1
Y2
Z2
Z5
chr8
0
20
40
60
80
100
120
140
chrEnd (Mbp)
CNV
10+ copies
5−9 copies
4 copies
3 copies
1 copy
0
CNVR
both
loss
gain

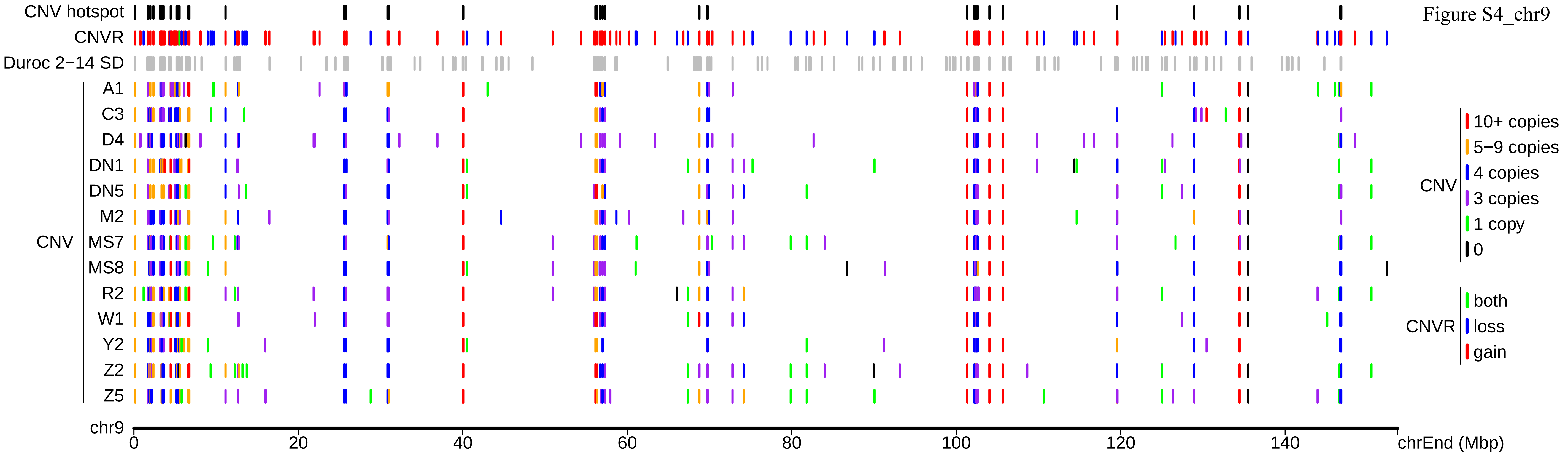
Figure S4_chr9
CNV hotspot
CNVR
Duroc 2−14 SD
A1
C3
D4
DN1
DN5
M2
CNV
MS7
MS8
R2
W1
Y2
Z2
Z5
chr9
0
20
40
60
80
100
120
140
chrEnd (Mbp)
10+ copies
5−9 copies
4 copies
3 copies
1 copy
0
both
loss
gain

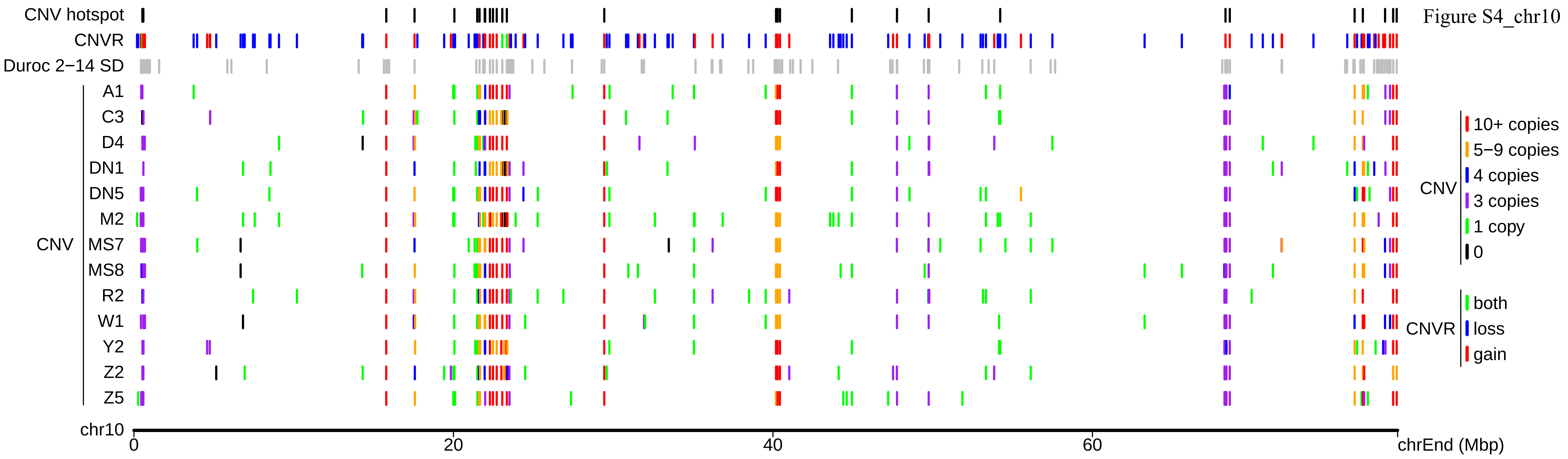
Figure S4_chr10
CNV hotspot
CNVR
Duroc 2−14 SD
A1
C3
D4
DN1
DN5
M2
CNV
MS7
MS8
R2
W1
Y2
Z2
Z5
chr10
0
20
40
60
chrEnd (Mbp)
10+ copies
5−9 copies
4 copies
CNV
3 copies
1 copy
0
both
CNVR
loss
gain

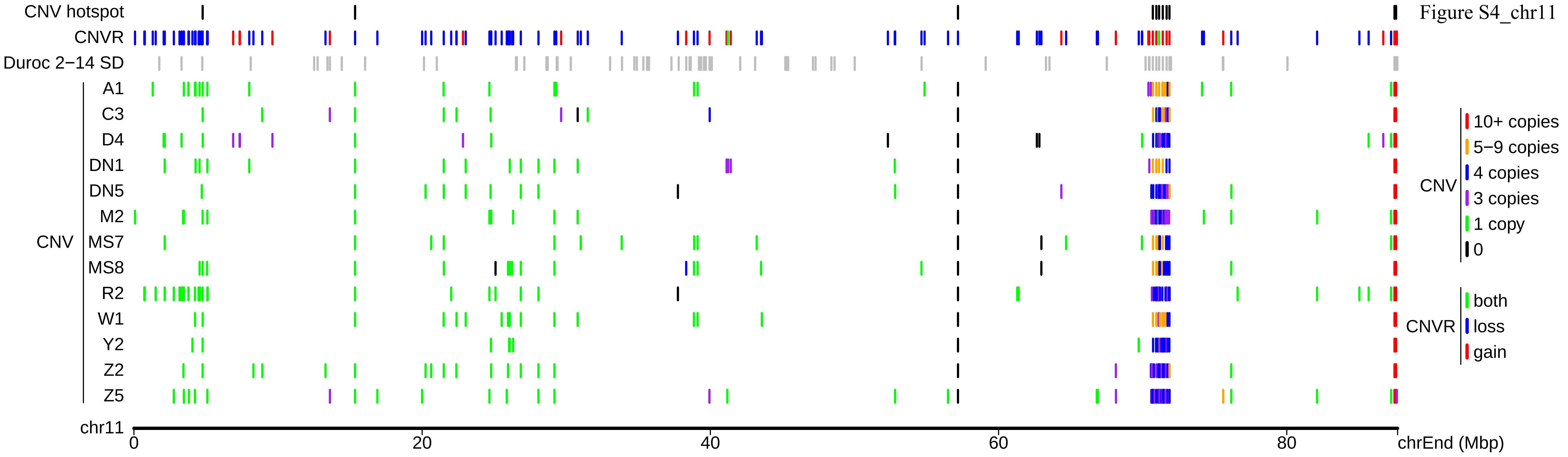

Figure S4_chr11
CNV hotspot
CNVR
Duroc 2−14 SD
CNV
A1
C3
D4
DN1
DN5
M2
MS7
MS8
R2
W1
Y2
Z2
Z5
chr11
0
20
40
60
80
chrEnd (Mbp)
CNV
10+ copies
5−9 copies
4 copies
3 copies
1 copy
0
CNVR
both
loss
gain

Figure S4_chr12

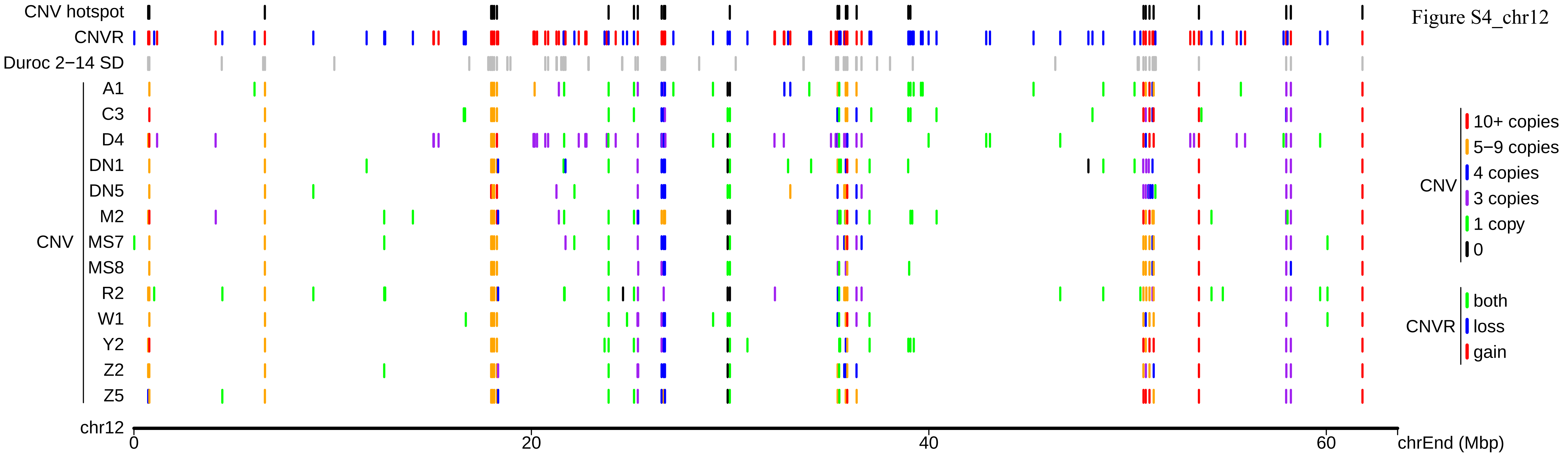

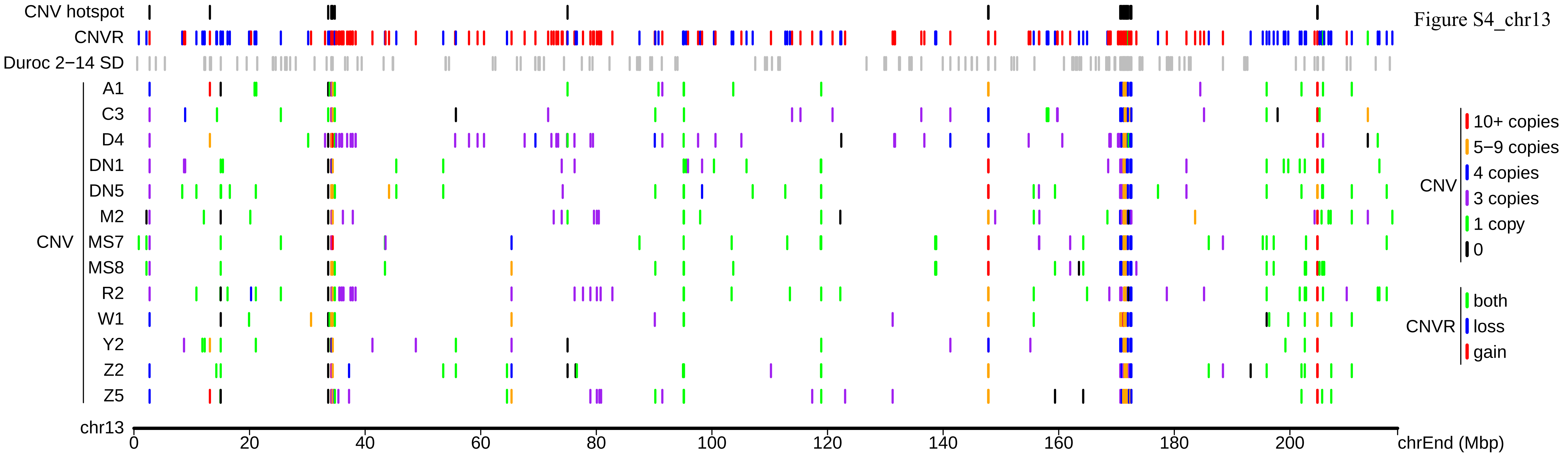

Figure S4_chr13
CNV hotspot
CNVR
Duroc 2−14 SD
A1
C3
D4
DN1
DN5
M2
MS7
MS8
R2
W1
Y2
Z2
Z5
CNV
10+ copies
5−9 copies
4 copies
3 copies
1 copy
0
CNVR
both
loss
gain
chr13
0
20
40
60
80
100
120
140
160
180
200
chrEnd (Mbp)

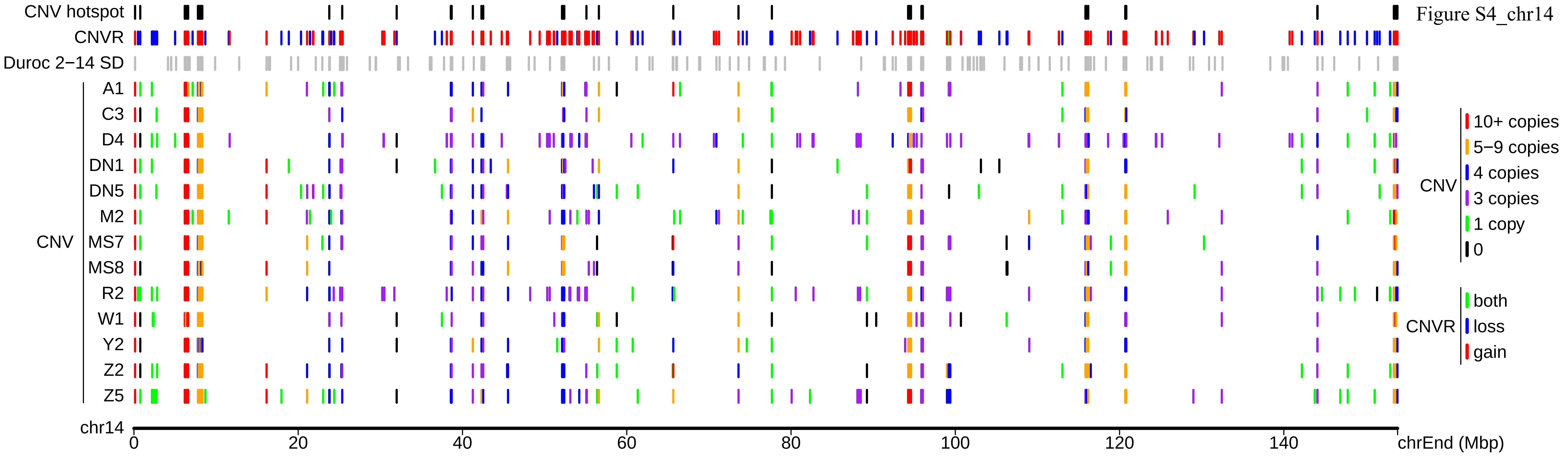
Figure S4_chr14
CNV hotspot
CNVR
Duroc 2−14 SD
A1
C3
D4
DN1
DN5
M2
MS7
MS8
R2
W1
Y2
Z2
Z5
CNV
chr14
0
20
40
60
80
100
120
140
chrEnd (Mbp)
10+ copies
5−9 copies
4 copies
3 copies
1 copy
0
CNV
both
loss
gain
CNVR

Figure S4_chr15

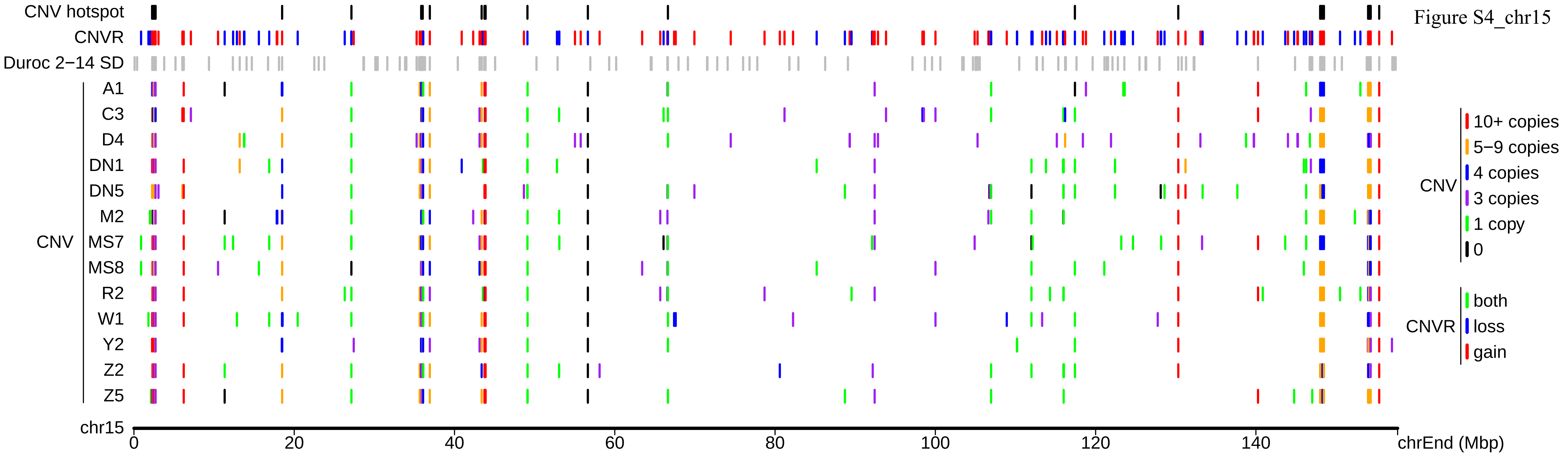

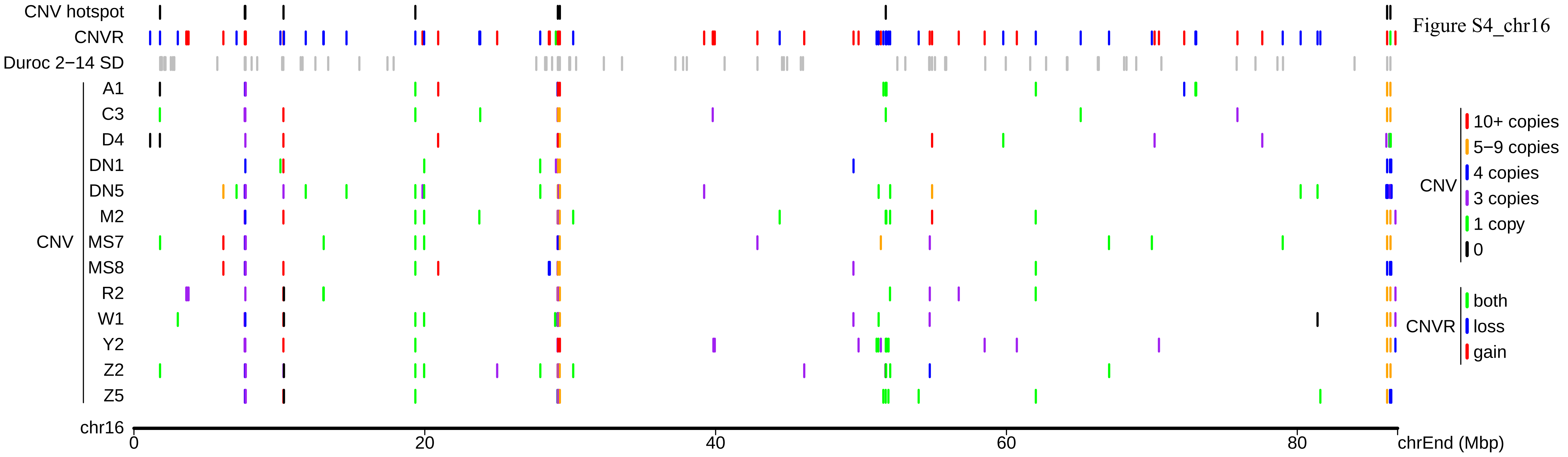
Figure S4_chr16
CNV hotspot
CNVR
Duroc 2−14 SD
A1
C3
D4
DN1
DN5
M2
MS7
MS8
R2
W1
Y2
Z2
Z5
CNV
chr16
0
20
40
60
80
chrEnd (Mbp)
CNV
10+ copies
5−9 copies
4 copies
3 copies
1 copy
0
CNVR
both
loss
gain

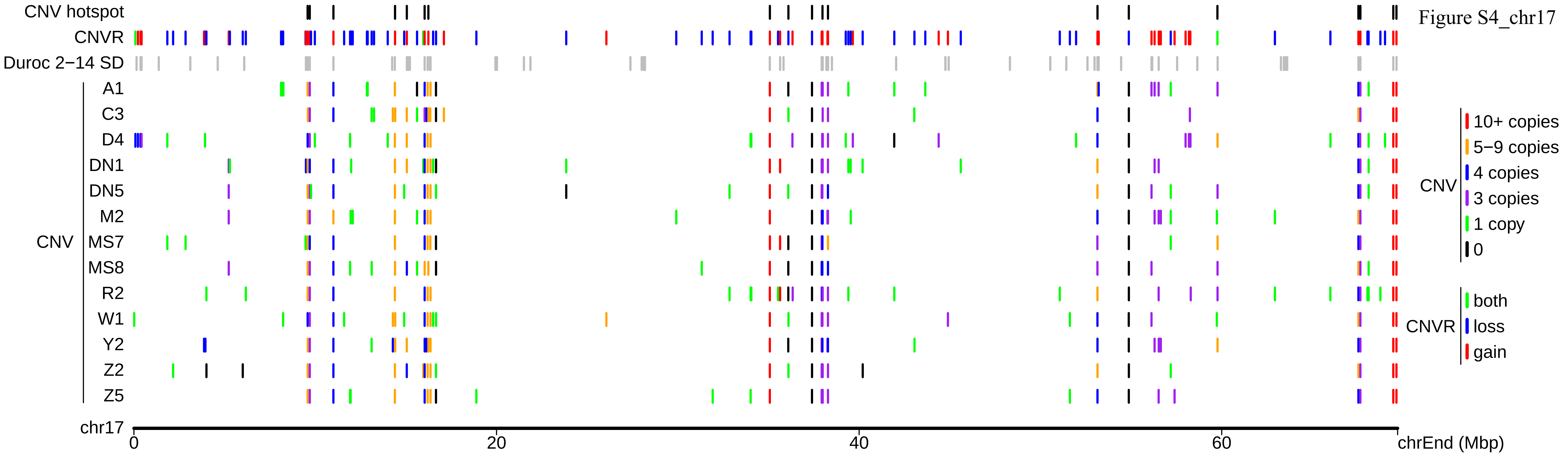

Figure S4_chr17

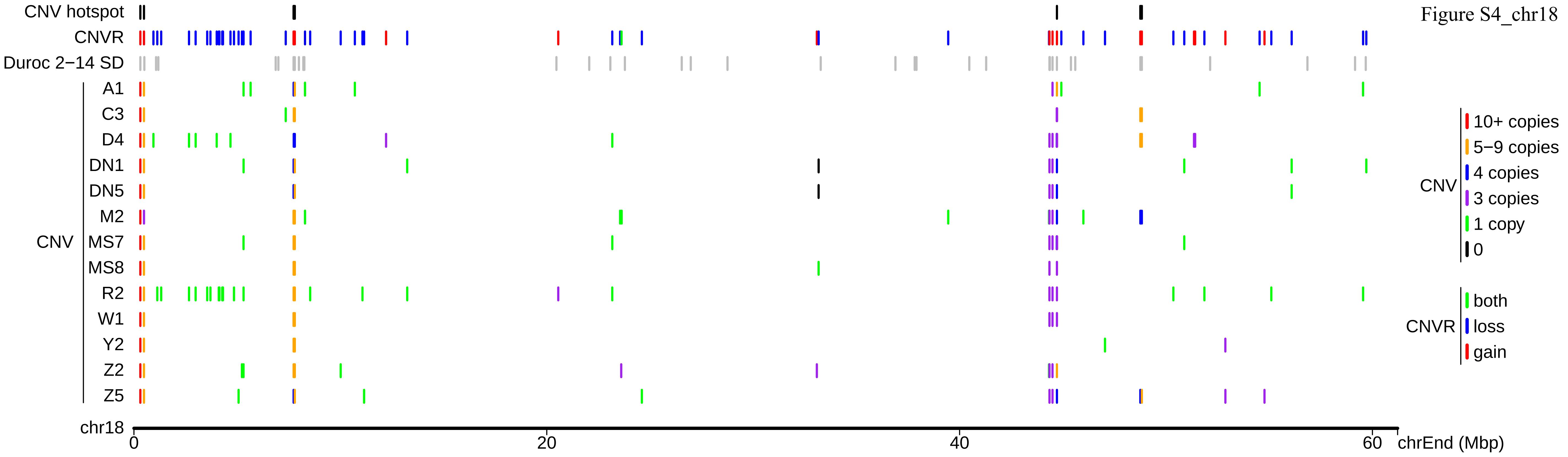

Figure S4_chr18
CNV hotspot
CNVR
Duroc 2−14 SD
A1
C3
D4
DN1
DN5
M2
CNV
MS7
MS8
R2
W1
Y2
Z2
Z5
chr18
0
20
40
60
chrEnd (Mbp)
10+ copies
5−9 copies
4 copies
CNV
3 copies
1 copy
0
both
CNVR
loss
gain

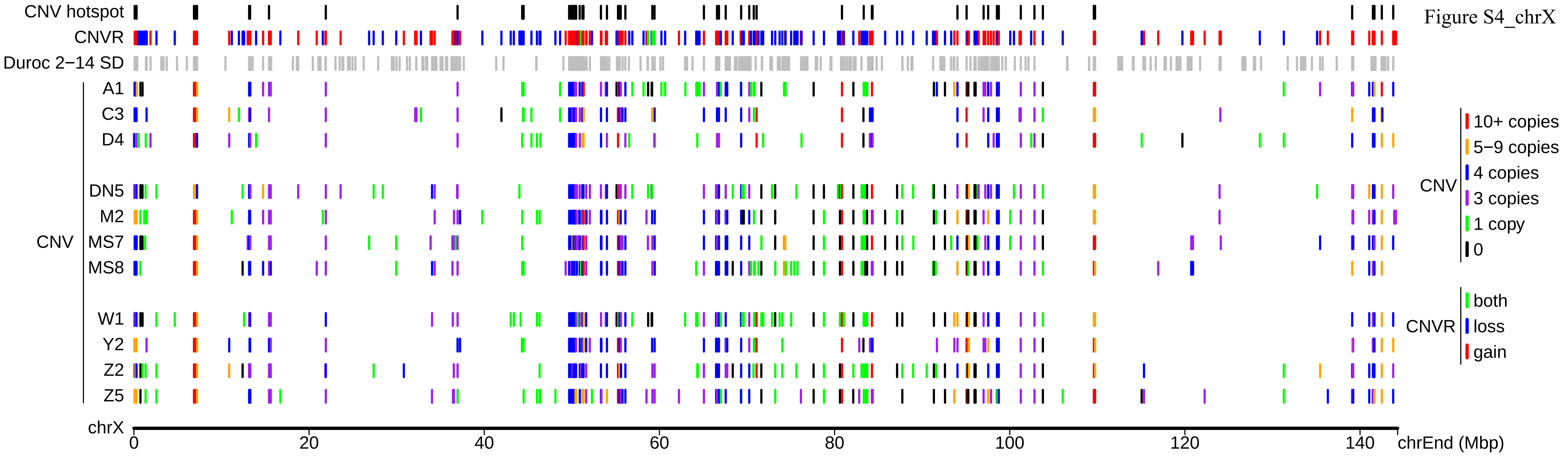
Figure S4_chrX
CNV hotspot
CNVR
Duroc 2−14 SD
A1
C3
D4
DN5
M2
MS7
MS8
W1
Y2
Z2
Z5
CNV
10+ copies
5−9 copies
4 copies
3 copies
1 copy
0
CNV
both
loss
gain
CNVR
chrX
0
20
40
60
80
100
120
140
chrEnd (Mbp)

Figure S5

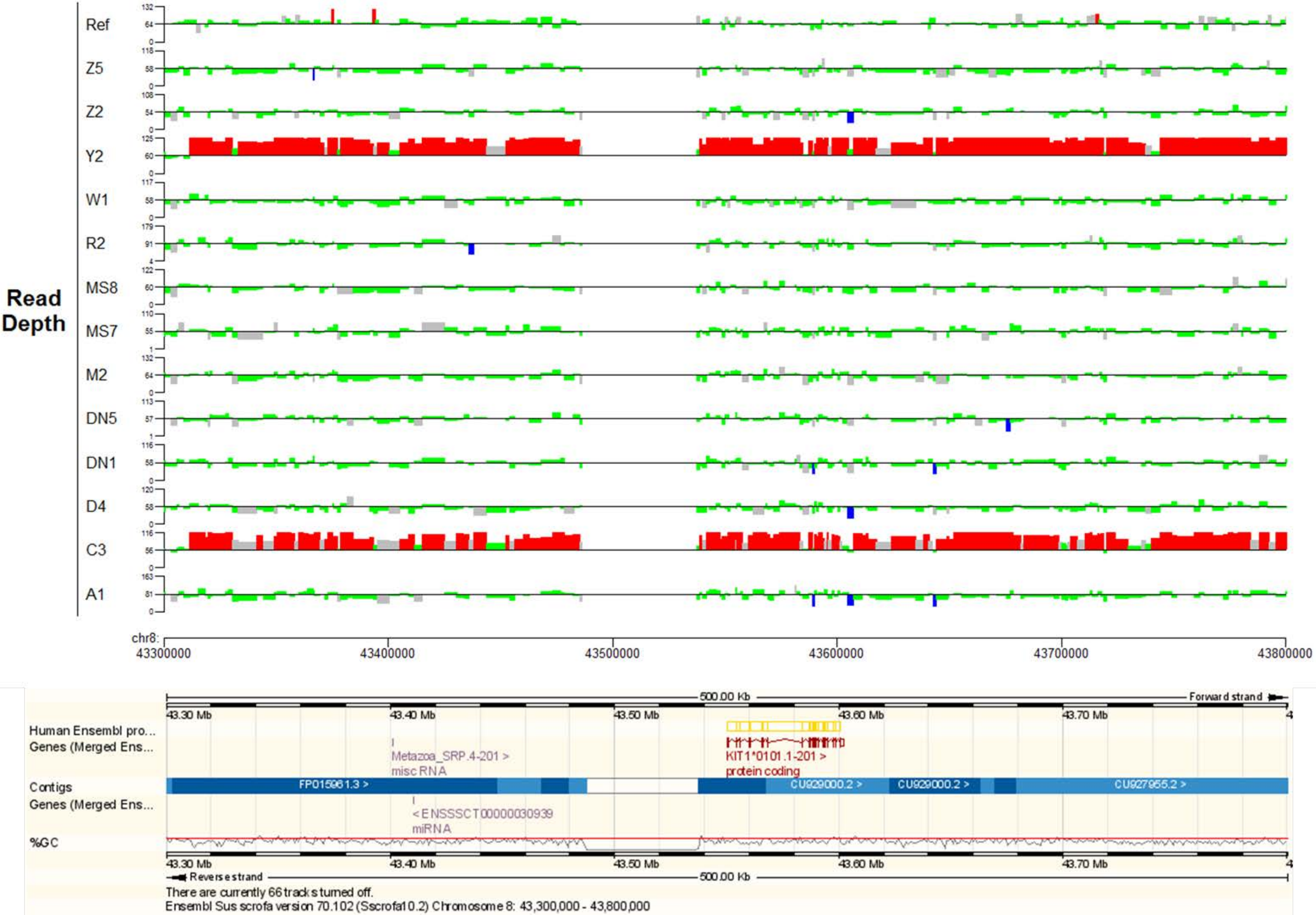

Read Depth
Ref
Z5
Z2
Y2
W1
R2
MS8
MS7
M2
DN5
DN1
D4
C3
A1
chr8:
43300000
43400000
43500000
43600000
43700000
43800000
500.00 Kb
Forward strand
43.30 Mb
43.40 Mb
43.50 Mb
43.60 Mb
43.70 Mb
Human Ensembl pro...
Genes (Merged Ens...
Metazoa_SRP.4-201 >
misc RNA
KIT1*0101.1-201 >
protein coding
Contigs
FP015961.3 >
CU929000.2 >
CU929000.2 >
CU927955.2 >
Genes (Merged Ens...
< ENSSSCT00000030939
miRNA
%GC
Reverse strand
There are currently 66 tracks turned off.
Ensembl Sus scrofa version 70.102 (Sscrofa10.2) Chromosome 8: 43,300,000 - 43,800,000

Figure S6

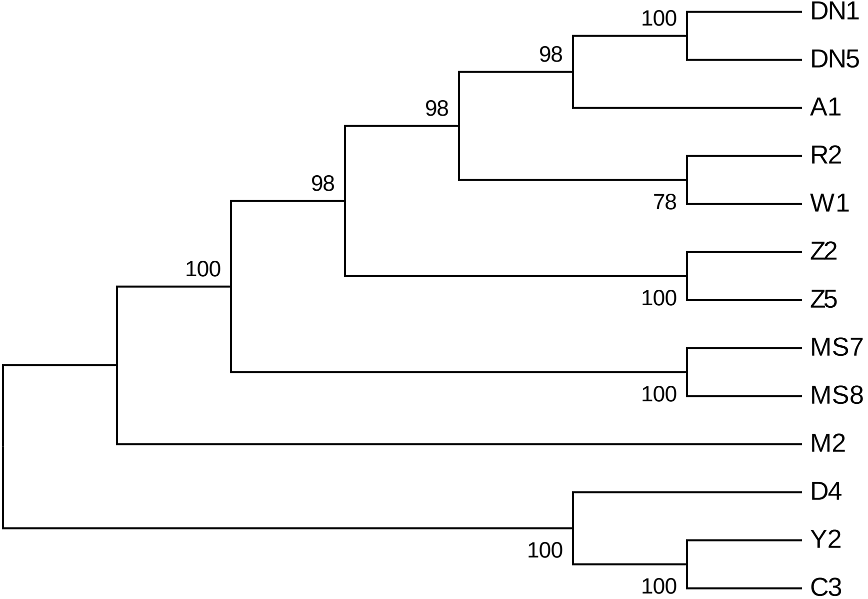

DN1
DN5
A1
R2
W1
Z2
Z5
MS7
MS8
M2
D4
Y2
C3
100
98
98
98
78
100
100
100
100
100
