# Supplementary material for: Global copy number analyses by next generation sequencing provide insight into pig genome variation
Source: BMC Genomics. 2014 Jul 14;15(1):593. doi: 10.1186/1471-2164-15-593 (PMC4111851; doi:10.1186/1471-2164-15-593)
Supplement: Supplementary file 7 — Additional file 7: Description: The file contains supplementary methods. (DOC 206 KB) [file 12864_2014_6278_MOESM7_ESM.doc]

**Supplementary Methods**

**Section 1**

**1 Developing an enhanced strategy in WSSD analyses**

For clearness, take an extreme example to explain how the fragmentation effect occurs. Hard masking may generate fragmented non-N genomic sequences like a 10 kbp sequence ([ATCG]{50}N{50}){100}. According to the original criteria in determining the three windows by mrCaNaVaR, this 10 kbp sequence may contain i) a long sliding window with the size of 5 kbp of non-masked characters as well as the sliding size of 1 kbp of any characters, ii) nine short sliding windows with the size of 1 kbp of non-masked characters and sliding size of 1 kbp of any characters and iii) five non-overlapping copy windows with size of 1 kbp of non-masked characters. It is clear herein that no any 100 bp reads can be aligned with this 10 kbp sequence within these three types of windows. Under such situation, mrCaNaVaR counts zero of RD for each of them whatever the genomic interval is actually duplicated or not, leading to substantial bias in duplication/deletion calls, *i.e.*, the fragmentation effect defined herein. Hence false positive deletions would frequently happen due to the fragmentation effect. Furthermore, fragmentation effects may vary among different genomic regions because of different degrees of fragmentation for different genomic sequences masked. This accordingly increases the variance of RD amonglong windows (LWs), short windows (SWs) and copy windows (CWs) and also makes a more heterogeneous background for RD calculation for each of these sliding windows, leading to the substantial decreases of sensitivity and specificity of duplication/deletion detection as well as accuracy of copy number estimation.

To reduce potential fragmentation effects, we modified mrCaNaVaR to optimize the way in defining the three types of windows, i.e., long window, short window and copy window. Specifically, the sizes of windows are based on the number of unique hits where short-reads can be forward aligned with the reference sequence rather than the accumulative counts of non-masked characters employed in the original mrCaNaVaR. In this study, for thereal IlluminaNGS dataand the short-read simulation data, the size sets for long sliding windows, short sliding windows and copy windows were determined as the genomic regions including 2,000, 400 and 400unique valid hits in the process of short-read alignments respectively,while for WSSD detection of the reference genome, these three values were set as 3,000, 600 and 600 respectively. In addition, the corresponding sliding step length was 1 kbp of any characters for both LW and SW for all datasets.

**2 The WGAC method for SD detection in the reference genome of swine**

**WGAC analyses**

WGAC is an assembly-dependent approach based on the BLAST strategy to detect paralogous stretches of sequence. The specific WGAC analyses in present study for the SD detection of the pig reference genome were performed in terms of three analytical steps as follows.

Firstly, Sscrofa 10.2 was masked using RepeatMasker open-3.3.0 (http://repeatmasker.org) and the pig Repbase libraries (version: 20120418) . The genome sequence was broken up into 400-kb segments, and the masked bases were then excised from the genome sequence. We detected seeding local alignments between the segments with the length ≥250 bp and identity ≥88%.The program LASTZ was then run to compare each 400kb segment to itself to detect local alignments with length ≥200bp and identity ≥88%.

Secondly, excised high-copy repeats were reinserted into each pairwise alignment, and then end trimming was performed for all the alignments to better define end points and to extend the alignments to repeat regions with the use ofoptimal global alignment program ALIGN in the FASTA package (version: 21.1.1) . After end trimming, ALIGN was used to generate optimal global alignments and calculate relevant statistics. Two pairwise alignments will be joint if both query and subject have gap sizes of <10 kb and at least one of them has a gap size of < 40 bp. Global alignments that equal or exceed the threshold of 1000 bases aligned and ≥90% identity (gaps excluded) were retained.

Finally, to better define the true duplications, prevalent interspersed high-copy repeats were removed on the basis of two criteria by custom Perl programs: (1) the duplications equal or more than 50 copies present in the genome; (2) with a distribution of equal or more than three placed chromosomes.

**WSSD analyses**

Besides WGAC, WSSD method with our enhanced strategy aforementioned was adopted to detect SDs. Unlike the precious studies , we here used NGS data instead of Sanger sequencing data to detect whole-genome SDs based on the idea of . Specifically, sequence repeats of Sscrofa 10.2 were masked using RepeatMasker (http://repeatmasker.org), Tandem Repeats Finder and WindowMasker. After masking, the portion of nonmasked and nongapped regions on the 20 chromosomes (1-18, X, Y) of pig genome reached 40.8%. Unplaced scaffolds were elided for further analyses.

Since the original NGS data with RD coverage of ~6x of Ducoc 2-14 contained two different types of sequence reads in length of 108 bp and 54 bp respectively, we split all 108 bp reads into two nonoverlapping 54 bp reads for consistency of read length. Subsequently, mrsFAST was employed to map short reads to the masked Sscrofa 10.2, allowing for up to 3 mismatches (*i.e.*, ~94.4% sequence identity). Approximately 21.5% of the raw reads were mapped to the masked genome with an average mapping count of 1.16 per read. The original program mrCaNaVaR was modified based on our enhanced CNV calling strategy and was then run to calculate GC% corrected RD and CN under the framework of additive GC correction mode. Initial SD calls were conducted if at least 6 of 7 sequential long sliding windows showed an excess RD value (>mean+3×STDEV). End points of calls were then refined using RD values of SW with a cutoff value of mean+2×STDEV. The absolute copy number of SD calls is predicted as CN median of all copy windows within them. Only intervals with CN>2.5 were considered as positive calls within 2-copy normal regions (***e.g.***, X chromosome of the female genome and the autosomes). Finally, all SD calls >10kb in length were kept in the final dataset.

**3 Detection of duplication/deletion for re-sequenced individuals**

Firstly,mrsFAST was used to align short reads to the masked Sscrofa 10.2, allowing up to 5 mismatches to attain 95% sequence identity. Then, mrCaNaVaRwas run to calculate GC% corrected RD values and copy numbers using re-defined windows in additive GC correction mode.

We set the specific RD criteria herein as: If at least 6 of 7 sequential long sliding windows showed an RD value that significantly exceeded the RD average (>mean + 4×STDEV), the interval was called as a SD. The SDs were refined using short sliding windows based on the RD threshold (>mean + 2×STDEV). Similarly, at least 6 of 7 sequential long sliding windows that showed a significantly reduced RD value (<mean - 4×STDEV) were called as a deletion. The deletions were also refined using short sliding windows based on the RD threshold (<mean -2×STDEV). Other steps were the same as described in WSSD method used in SD map construction.Compared to autosomes, less information can be made use of for SD/deletion identification on sex chromosomes in male pigs, so the sex chromosomes in male pigs were ignored to ensure the detection reliability.

As previously reported , we mimicked NGS data of the reference genome to address the issue of short-read artifacts. Specifically, the sequence of Sscrofa 10.2 was broken into 100 bp reads with sliding size of 1 bp, which generated NGS data with RD of ~100×. MrsFAST was run to map these simulated reads back to the masked Sscrofa 10.2. SD intervals were called with the same parameter set for the analyses of real IlluminaNGS data. Comparing SDs of the simulated data to the identified SDs (WGAC + WSSD) for the reference genome, we treated any intervals as short-read “artifacts” if they did not agree with the identified SDs. Finally, these short-read artifacts, covering a total length of 17.2Mbp of autosome region and 1.6Mbpof the X chromosome region, were excluded from the SD predictions for all 13 samples.

**4 Validation of pig CNVs using aCGH and** **qPCR**

**aCGH validation**

In aCGHanalyses, the individual D4 (Duroc) was used as the reference, while the other 12 individuals as the test samples. Spatial correction and data normalization were performed using the NimbleScan Version 2.4 software (Roche-NimbleGen). We then employed the method as reported previously to validate the RD called duplications/deletions byaCGH data. Firstly we sifted out all RD predicted CNV intervals with the sizes greater than 20kbp as well as containing <80% common repeat contents.

For each pair of samples, *i.e.*, the reference D4 and each of other 12 test samples, we defined two novel genomic regions based on the filtered CNV intervals:i) a subinterval shared by two CNVs each belonging to respective paired samples, ii) a region covered merely by a CNV interval for either of paired samples while being normal copy number region in the other sample. For each of these intervals, we computed the log_2_ ratio of estimated CN of test sample to that of the reference as well as the averaged log_2_ ratios of all aCGH probes fell into the interval. The Pearson’s correlationcoefficient between the log_2_ CN ratios and aCGH probe log_2_ ratios was then calculated, which can act as the indicator of the conformity between RD and aCGH predictions.

**qPCR confirmation**

In qPCR experiments, primers (Table 1) were designed with the Primer3 web tool (http://frodo.wi.mit.edu/primer3/). To ensure the same amplification efficiencies between target and control primers, the PCR efficiencies for all primers used in the study were required to be 1.95-2.10. All qPCR were carried out using LightCycler® 480 SYBR Green I Master on Roche LightCycler® 480 instrument following the manufacturer’s guidelines and cycling conditions. The reactions were carried out in a 96-well plate in 20μl volume, containing 10μl Blue-SYBR-Green mix, 1μl forward and reverse primers (10pM/μl) and 1μl20ng/μl genomic DNA. All RT-PCRs were runin duplicates. The second derivative maximum algorithm included within the instrument software was used to determine cycle threshold (Ct) values for each region and the average of Ct value of two replications of each sample was calculated and normalized against the control genewith the assumption that there are two copies of DNA segment in the control region. For each CNVR to be validated, a value from the formula 2×2^-ΔΔCt^ was calculated foreach individual.For each of detected CNVs, an estimate of CN>2.5indicates theindividual is in gain status, a CN<1.5 denotes the loss status, and the value between 1.5 and 2.5 value demonstrates the normal status.

Particularly, to systematically assess the performance of RD-based CNV calling, we defined four different experimentscenarios in qPCRvalidation: (1) the number of true positive findings (TP), which denoted as the positive detections called by the RD method as well as validated by qPCR; (2) the number of true negative findings (TN), meaning the negative detections determined by the RD method as well as confirmed by qPCR;

(3) the number of false positive detections (FP) with the meaning of the CNV findings called by the RD method while not confirmed by qPCR; and (4) the number of false negative detections (FN), denoting as the negative CNV findings determined by the RD method while not confirmed by qPCR.

According to these scenarios, we proposed three different evaluation criteria to comprehensively assess the performance of the RD method, including: (1) the overall agreement rate calculated by (TP+TN)/(TP+TN+FP+FN); (2) the detection power of the RD method, expressed as TP/(TP+FN); and (3) the positive prediction rate of the RD method, which defined as TP/(TP+FP).

**Section 2**

**Simulation analyses for validation of the enhanced strategy in WSSD**

**Simulation Scheme**

**Short read simulation**

Weselected chromosome 11 from the reference genome assembly of Sscrofa 10.2as the basis for short read simulation, considering chromosome 11 has a moderate sequence length and involves segmental duplication (SD) intervals with a relatively high average size(10.6 kbp)derived fromourinitial WGAC analyses.

We simulated next-generation sequencing (NGS) data under various scenariosto extensively evaluateperformance of the enhanced WSSD analyses. Specifically, the sequence of chromosome 11 was broken into equal-length reads with sliding size of 1bp to separatelymimic three types of sequencing reads with distinct sizes of 36bp, 100bp and 200bp, generating corresponding reads datasets with depth of coverage of ~36×, ~100× and ~200×.From each dataset, we randomly sampled different amount of read data to construct three reads subsets with depth of coverage of ~5×, ~10× and ~30×, respectively.Through combination of different levels of reads length and depth of coverage, the simulation of short read finally included nine different scenarios.

**Determining true SDs and****benchmarking for performance evaluation**

All 1,234intrachromosomalpairwise alignments on chromosome 11 from our initial WGAC analyses were picked out in the simulation analyses.Among these raw output of pairwise alignments, we removed the pairwise alignments with <95% identity, and the remaining alignments were then employed to determinethe true SD regions on chromosome 11. We derived duplicated regions by merging overlapping alignments and then defined 2-copy normal regions as those outside the duplicated regions.These SD regions and 2-copy normal regions were considered as benchmarking for performance evaluation of different RD analytical strategies.

Herein we adopted two commonly-used criteria, *i.e.*,detection power and proportion of false positivedetection among all SD findingsto evaluate performance of our enhanced as well as the original WSSD analyses through examining whether a duplicated region was successfully identified and whether a normal 2-copy region was falsely detected as a SD region.

Furthermore, within the duplicated regions, we speciallyfocused on those pairwise alignmentswith no overlapping with anyother alignments, which can be clearly considered as 4-copy SD regions. The 4-copy SD regions and the 2-copy normal regions were considered as the chromosome regions with true copy number known, which can be further used for assessing accuracy of copy number estimates bydifferent methods through calculatingaverage and standard deviation of copy numbers within these copy number known regions.

**Short read mapping and SD detection**

As described in the main text, we used mrsFAST for mapping simulated short reads with the hamming distance being 5% of the read length (*i.e.* 2 for 36 bp reads, 5 for 100 bp reads and 10 for 200 bp reads), and further ran mrCaNaVaRfor SD detection and absolute copy number estimation.

For the original method, the sizes of three types of sliding windows (*i.e.*long windows, short windows and copy windows) were set as default in mrCaNaVaR, *i.e.* 5,000 bp of non-masked characters for long windows, 1,000bp of non-masked characters for short windows and 1,000 bp of non-masked characters for copy windows;while inour modified method, we empiricallytuned in the sizes of the three windows to ensure the resulting number of each windowapproximatelyequal to that generated by the original method (see Table 2 for setting of window sizes). Accordingly, the sequence read amount per window defined by the original and our enhanced method can be largely kept at a same level, such that the performance comparison between two analytical strategies can be conducted under the same basis,bringing a convincing evaluation in the analyses.

**Simulation Results**

**Comparison of copy number estimation accuracy**

We calculated means and standard deviations(Std) of copy numbersof copy windows within 2-copy normal regions and 4-copy SD regions, respectively. As shown in Tables 3 and 4, a lower Stdof CN estimates is always foundin the modified method across all simulated scenarios, reflecting higher accuracy of CN estimates by our enhanced strategy than that by the original method. This trend is becoming more obvious with the increase of simulated read length.

Wealso calculated the proportion of copy windows with false copy number estimates in the process of SD calling. A false copy number estimate is defined herein as the copy number estimate of <1.5 or >2.5 for copy windowswithin 2-copy normal regions, as well as of <3.5 or >4.5 for copy windows within 4-copy SD regions.Compared with the original method, the modified method predicted much less proportion of copy windows with false copy number estimates almost under all scenarios, reaching more than 10-fold decreasein someof simulation scenarios compared with the original method.

**Comparison of detection power and** **proportion of false positive detections among all SD findings**

As shown in Table 5, the modified method predicts much less deletions than the original method almost in each scenario, indicating the modified method has a better performance in controlling false positive detection of segmental deletions. This is because all deletion findings are impossibly present in the simulation analyses and these identified deletions are definitely false positive. Additionally, compared with the original method, the modified method has an improved detection power across all scenarios, especially for long sequence read data with length of 100 bp and 200 bp.Moreover, we further assessed proportion of false positive detections among all SD findings and the simulation results showed that the modified method generallypredicted less false positive SDs than the original method. This advantage is becoming more visible with the increase of length of simulated read data.

**Conclusion**

Through redefining three different windows using our proposed strategy, we can improve the performance of the WSSD analyses in CNV calling. The gain in performance includes the increases of accuracy of CN estimates and detection power, as well as the decreases of the proportion of false positive detections among all SD findings, especially for detection of deletions.

**References**

1. Jurka, J., Kapitonov, V.V., Pavlicek, A., Klonowski, P., Kohany, O. and Walichiewicz, J. (2005) Repbase Update, a database of eukaryotic repetitive elements. *Cytogenet Genome Res*, **110**, 462-467.

2. Harris, R.S. (2007), The Pennsylvania State University.

3. Pearson, W.R. and Lipman, D.J. (1988) Improved tools for biological sequence comparison. *Proc Natl Acad Sci U S A*, **85**, 2444-2448.

4. Bailey, J.A., Church, D.M., Ventura, M., Rocchi, M. and Eichler, E.E. (2004) Analysis of segmental duplications and genome assembly in the mouse. *Genome Res*, **14**, 789-801.

5. Bailey, J.A., Gu, Z., Clark, R.A., Reinert, K., Samonte, R.V., Schwartz, S., Adams, M.D., Myers, E.W., Li, P.W. and Eichler, E.E. (2002) Recent segmental duplications in the human genome. *Science*, **297**, 1003-1007.

6. Alkan, C., Kidd, J.M., Marques-Bonet, T., Aksay, G., Antonacci, F., Hormozdiari, F., Kitzman, J.O., Baker, C., Malig, M., Mutlu, O. *et al.* (2009) Personalized copy number and segmental duplication maps using next-generation sequencing. *Nat Genet*, **41**, 1061-1067.

7. Benson, G. (1999) Tandem repeats finder: a program to analyze DNA sequences. *Nucleic Acids Res*, **27**, 573-580.

8. Morgulis, A., Gertz, E.M., Schaffer, A.A. and Agarwala, R. (2006) WindowMasker: window-based masker for sequenced genomes. *Bioinformatics*, **22**, 134-141.

**Table legends**

Table 1. Information of qPCR primers for CNV validation

Table 2. Specified window sizes and resulting window numbers

Table 3. Summary of copy number estimates of copy windows in 2-copy normal regions

Table 4. Summary of copy number estimates of copy windows in 4-copy SDs

Table 5. Summary of predicted deletions/SDs using RD methods in different scenarios

Table 1.Information of qPCR primers for CNV validation

| CNVR Information | | | | | Primer Informaiton | | | | | |
| --- | --- | --- | --- | --- | --- | --- | --- | --- | --- | --- |
| CNVR # | Chr. | Start | End | Type | Primer Name | Primer_F | Primer_R | Start | End | Length |
| 147 | chr1 | 224908948 | 224929711 | gain | RD13A-4 | CCTTCTGGACCTGGTTGCC | CCTGCCTCAGACCCACAGC | 224913013 | 224913158 | 146 |
|  |  |  |  |  | RD13B-3 | AGGCTTGACTTGGGTTGCTTC | GAGGACACATCATAGAGTTTGGTAGAG | 224920852 | 224920965 | 114 |
| 203 | chr1 | 284427746 | 284523631 | gain | CGH1-1 | GGGCGATGATGGACAGTAAGG | GTGGATGGAGCAGGAGGTGT | 284441084 | 284441237 | 154 |
|  |  |  |  |  | Gene2-2 | CCATAAATTGGAGGGTGAGATG | AGTTGTAAGTGGGAAACGGGTG | 284448182 | 284448284 | 103 |
| 302 | chr10 | 22872897 | 23243756 | both | LD1-2 | CCAAATAGGAGGTGGCTCTGT | AGCAGGTATAAGGCAAGGGTTC | 23204716 | 23204883 | 168 |
|  |  |  |  |  | LD1-3 | TTACGAAGTTCCGTGCTGCTAC | GCCTTTACAATATCCTGCTCTGC | 23205934 | 23206060 | 127 |
| 477 | chr11 | 62572158 | 62742807 | loss | F1-2 | AGTGACTGCTGACACGAGAGG | ATGCTGGGCACATTATTGGAC | 62718209 | 62718355 | 147 |
|  |  |  |  |  | F1-3 | ATGCCTTCTTTTATCCAACTCAC | TTCCCCACTCTCTCAAATACCTC | 62735125 | 62735323 | 199 |
| 479 | chr11 | 62943791 | 63001040 | loss | RD2A-2 | CCTTCCTCCACTTGCAGACAGAG | GGGATATGGGATTTAACGACCTG | 62948181 | 62948324 | 144 |
|  |  |  |  |  | RD2B-4 | GGTCCCTCCTGACAGTATGGC | CACCTGGACTTGGTCTCATCAC | 62992983 | 62993132 | 150 |
| 490 | chr11 | 70554697 | 70853929 | gain | J2-1 | GGTTATCTGGGGTGTGGAGGA | TGGAGGGTCTGAGAACTTTGG | 70678244 | 70678368 | 125 |
| 621 | chr12 | 57957324 | 58013169 | gain | RD11A-1 | AGAGCAGCAGGCAACAAAGTC | CCATTGGGTTAAGTGTATAAATCAGTC | 58008243 | 58008387 | 145 |
|  |  |  |  |  | Gene16A-3 | TAATCTTCGCCTTGACCTTTCC | AGGGTGTTGACTTTGTCCTCTTCT | 57973945 | 57974105 | 161 |
| 623 | chr12 | 58204324 | 58227964 | gain | RD11B-2 | CGTTCATCCCACAGGACACC | TGTTCTGCGACTTTCCTACTCC | 58226477 | 58226648 | 172 |
|  |  |  |  |  | Gene16B-1 | GCATCGTCTGCCAAACCTTTC | TATCCGCATTCTTCCACATCG | 58206565 | 58206702 | 138 |
| 660 | chr13 | 34197467 | 34233307 | gain | CGH3-1 | GGATGATGGAGCGGGAGAAG | CCACTGCCACTAAATGCTTGTC | 34207669 | 34207871 | 200 |
|  |  |  |  |  | Gene9-4 | GCCAGGGTAGGAGGGTGTT | TTCTAGCGGGTGAAGCAGTG | 34209353 | 34209540 | 188 |
| 1456 | chr2 | 9806209 | 9868133 | gain | RD10A-4 | AGGATTTGCCTCCATTTGTGC | TACCCTGCCCTCTACTCATTTCTC | 9833907 | 9834075 | 169 |
|  |  |  |  |  | RD10B-1 | TCAATGTTCACCCTTGTTGTCG | CAGTAGATGTTCTGTGCCAGTCGT | 9849712 | 9849847 | 136 |
| 1538 | chr2 | 61317102 | 61368938 | both | R1-3 | TTTTGGATAGGATGGCGGACT | ATTGATGGGTATTGTTCTGGATGT | 61356969 | 61357128 | 160 |
| 2053 | chr4 | 126260104 | 126338355 | gain | Gene15B-3 | CATCTATTCTTACATTCTGGGACG | GGGCGAGCATAAATCCAACT | 126283963 | 126284117 | 155 |
|  |  |  |  |  | Gene15B-4 | AGCATCTATTCTTACATTCTGGGACG | AGTTGGATTTATGCTCGCCC | 126283961 | 126284117 | 157 |
| 2436 | chr7 | 24620712 | 24762578 | gain | RD14A-2 | CCCACCTCCCTTCCCTACCT | CCTTGCCCTTTGTTACCAGACC | 24684808 | 24684955 | 148 |
|  |  |  |  |  | RD14B-2 | TCATCCGCTCCGCCACAT | TACGATCAGGACGCCTACGAC | 24750533 | 24750665 | 133 |
| 2472 | chr7 | 50300711 | 50673922 | loss | D1-1 | TTGGGAAATGTTCAACTGTGTA | TCAATGGAATGAGGTAGGGTCT | 50374770 | 50374933 | 164 |
|  |  |  |  |  | D2-1 | TCATTGGGTCTACTTCCTTCTTCTTA | AGTTCATTCACCTATGCCTCTCTTAT | 50413778 | 50413967 | 190 |
| 2488 | chr7 | 59577619 | 59600227 | loss | LC1-1 | TGGAGGGAGGAGAAATGGATG | TGTGATGGATGTGGCAGAAGAG | 59581146 | 59581285 | 140 |
|  |  |  |  |  | LC1-2 | TGCTACCCATCGCTCCATAC | TGTGATCCAAGAACCCAAATCTC | 59586275 | 59586505 | 231 |
| 2596 | chr8 | 5730978 | 5748400 | loss | LC4-3 | GTGGACCCTTGACTCTTCGTGT | CAATGTGATGCCAATTTCTGCT | 5735212 | 5735390 | 179 |
| 2628 | chr8 | 43309551 | 43485585 | gain | CGH2-1 | CTGTTGGGAGCAGATGAGACC | GGGCGAAGGAAAGGATGTAAG | 43319160 | 43319303 | 144 |
| 2770 | chr9 | 11126204 | 11152941 | gain | RD8-2 | CGCCTACTTAGCGGTTTGGAC | GAAATGACTGGATGGTGGAGATG | 11131204 | 11131314 | 111 |
| 3068 | chrX | 91322641 | 91355147 | loss | CGH5B-1 | AGCACAGCGATGGCTCTACAAC | GCATTGCCTGAGTAAACATCAACAG | 91340081 | 91340211 | 131 |
|  |  |  |  |  | CGH6A-2 | ATTATCTTCATTGCCATTTGCCT | GGAATCTTTTCGGACCACAACAC | 91332745 | 91332918 | 174 |
|  |  |  |  |  | CGH6B-1 | AGCACAGCGATGGCTCTACAAC | CATTGCCTGAGTAAACATCAACAG | 91340081 | 91340210 | 130 |
| 3071 | chrX | 92590788 | 92623408 | loss | R2-2 | AGGAGCAAACGATGAAACGG | CACCTCAGGGCAAAGGACAC | 92592688 | 92592881 | 194 |
| 3104 | chrX | 115317616 | 115340965 | gain | RD4A-2 | AGAGTCAGGCTGTGGATTAAAGG | GGTTCTGGATAACTGAGTGGGTAG | 115321471 | 115321579 | 109 |

Table 2.Specified window sizes and resulting window numbers

| Window type^a^ | The original method | | The modified method^b^ | | | | | |
| --- | --- | --- | --- | --- | --- | --- | --- | --- |
|  |  |  | 36 bp read | | 100 bp read | | 200 bp read | |
|  | Window size | Window number | Window size | Window number | Window size | Window number | Window size | Window number |
| LW | 5000 | 76145 | 3500 | 76139 | 2000 | 75920 | 1000 | 74945 |
| SW | 1000 | 77785 | 700 | 77755 | 400 | 77646 | 200 | 77338 |
| CW | 1000 | 36568 | 700 | 36710 | 400 | 33080 | 200 | 23725 |

^a^LW, long window; SW, short window; CW, copy window.

^b^The unit of window size in the modified method is unique hit where short-reads can be forward aligned with the reference sequence.

Table 3. Summary of copy number estimates of copy windows in 2-copy normal regions

| Scenario | Read length | Depth of coverage | Copy number of copy windows -  Mean (Std) | | Proportion of copy windowswith false copy number estimates (%)^a^ | |
| --- | --- | --- | --- | --- | --- | --- |
|  |  |  | Original method | Modified method | Original method (<1.5 \| >2.5)^b^ | Modified method  (<1.5 \| >2.5)^b^ |
| 1 | 36 | 30 X | 2.043 (0.452) | 2.050 (0.397) | 2.1 | 3.3 | 0.1 | 2.5 |
| 2 | 100 |  | 2.008 (0.721) | 2.038 (0.381) | 23.3 \| 21.4 | 1.0 \| 2.8 |
| 3 | 200 |  | 2.353 (1.686) | 2.026 (0.594) | 36.8 \| 39.1 | 18.2 \| 17.6 |
| 4 | 36 | 10 X | 2.047 (0.470) | 2.053 (0.415) | 3.0 | 4.7 | 0.2 | 2.5 |
| 5 | 100 |  | 2.000 (0.754) | 2.041 (0.446) | 25.3 \| 22.3 | 5.1 \| 7.7 |
| 6 | 200 |  | 2.398 (1.816) | 2.007 (0.692) | 37.8 \| 39.5 | 23.9 \| 21.2 |
| 7 | 36 | 5 X | 2.050 (0.486) | 2.054 (0.435) | 4.3 | 6.4 | 0.6 | 3.2 |
| 8 | 100 |  | 2.000 (0.814) | 2.043 (0.526) | 28.0 \| 23.8 | 10.9 \| 13.6 |
| 9 | 200 |  | 2.591 (2.088) | 1.999 (0.836) | 37.5 \| 42.6 | 29.1 \| 24.2 |

^a^A false copy number estimate is defined herein as the copy number estimate of <1.5 or >2.5 for copy windows within 2-copy normal regions.

^b^The left of “|” indicates the proportion of copy windows with copy number estimate <1.5, and the right indicates the proportion of copy windows with copy number estimate >2.5.

Table 4. Summary of copy number estimates of copy windows in 4-copy SDs

| Scenario | Read length | Depth of coverage | Copy number of copy windows -  Mean (Std) | | Proportion of copy windows with false copy number estimates (%)^a^ | |
| --- | --- | --- | --- | --- | --- | --- |
|  |  |  | Original method | Modified method | Original method  (<3.5 \| >4.5)^b^ | Modified method  (<3.5 \| >4.5)^b^ |
| 1 | 36 | 30 X | 3.965 (0.634) | 3.955 (0.421) | 19.1 | 13.5 | 2.7 | 2.0 |
| 2 | 100 |  | 3.924 (1.401) | 3.893 (0.485) | 40.9 \| 32.2 | 17.2 \| 5.0 |
| 3 | 200 |  | 4.523 (3.393) | 3.776 (1.011) | 46.6 \| 42.0 | 43.1 \| 24.6 |
| 4 | 36 | 10 X | 3.968 (0.657) | 3.956 (0.459) | 20.6 | 13.9 | 4.5 | 2.3 |
| 5 | 100 |  | 3.918 (1.437) | 3.912 (0.607) | 42.0 \| 31.1 | 22.0 \| 13.3 |
| 6 | 200 |  | 4.563 (3.537) | 3.720 (1.144) | 47.3 \| 42.3 | 45.9 \| 25.3 |
| 7 | 36 | 5 X | 3.989 (0.687) | 3.971 (0.492) | 20.5 | 17.2 | 7.7 | 4.2 |
| 8 | 100 |  | 3.907 (1.495) | 3.913 (0.704) | 42.4 \| 33.0 | 26.4 \| 18.2 |
| 9 | 200 |  | 4.970 (3.956) | 3.733 (1.279) | 44.6 \| 45.6 | 46.3 \| 26.6 |

^a^A false copy number estimate is defined herein as the copy number estimate of <3.5 or >4.5 for copy windows within 4-copy SD regions.

^b^The left of “|” indicates the proportion of copy windows with copy number estimate <3.5, and the right indicates the proportion of copy windows with copy number estimate >4.5.

Table 5. Summary of predicted deletions/SDs using RD methods in different scenarios

| Scenario | Depth of coverage | Read length | # of calls >10 kbp  (del. | dup.) | | Detection power^a^ | | Proportion of false positive detections among all SD findings^b^ (%) | |
| --- | --- | --- | --- | --- | --- | --- | --- | --- |
|  |  |  | Original method | Modified method | Original method | Modified method | Original method | Modified method |
| 1 | 30 X | 36 | 13 | 343 | 0 | 361 | 0.975 | 0.992 | 5.0 | 5.8 |
| 2 |  | 100 | 7 \| 230 | 0 \| 329 | 0.623 | 0.969 | 7.8 | 4.0 |
| 3 |  | 200 | 6 \| 188 | 0 \| 241 | 0.285 | 0.782 | 16.0 | 6.2 |
| 4 | 10 X | 36 | 7 | 336 | 0 | 358 | 0.972 | 0.989 | 4.5 | 5.3 |
| 5 |  | 100 | 4 \| 229 | 7 \| 314 | 0.592 | 0.947 | 7.0 | 3.8 |
| 6 |  | 200 | 5 \| 200 | 1 \| 218 | 0.307 | 0.648 | 16.0 | 6.9 |
| 7 | 5 X | 36 | 8 | 330 | 0 | 347 | 0.958 | 0.983 | 4.2 | 4.6 |
| 8 |  | 100 | 3 \| 224 | 2 \| 297 | 0.542 | 0.930 | 6.3 | 4.4 |
| 9 |  | 200 | 4 \| 209 | 1 \| 187 | 0.285 | 0.427 | 19.6 | 9.6 |

^a^Detection power represents the proportion of true positive findings in all true segmental duplications of >10 kbp in length. A true SD with its length of >60% identified by SD findings is considered as true positive finding.

^b^Only consider SD findings with interval of >10kbp. A false positive detection denotes an SD call with its length of >80% falling in a 2-copy normal region.
